# Engineering the Electronic Interaction between Atomically Dispersed Fe and RuO_2_ Attaining High Catalytic Activity and Durability Catalyst for Li‐O_2_ Battery

**DOI:** 10.1002/advs.202205975

**Published:** 2023-01-22

**Authors:** Zheng Lian, Youcai Lu, Shaoze Zhao, Zhongjun Li, Qingchao Liu

**Affiliations:** ^1^ Green Catalysis Center and College of Chemistry Zhengzhou University Zhengzhou 450001 P. R. China; ^2^ State Key Laboratory of Pulp and Paper Engineering South China University of Technology Guangzhou 510641 P. R. China

**Keywords:** atomically dispersed Fe, electronic metal‐support interaction, Li‐O_2_ batteries, Ru‐O‐Fe_1_ structure

## Abstract

It is significant to develop catalysts with high catalytic activity and durability to improve the electrochemical performances of lithium‐oxygen batteries (LOBs). While electronic metal‐support interaction (EMSI) between metal atoms and support has shown great potential in catalytic field. Hence, to effectively improve the electrochemical performance of LOBs, atomically dispersed Fe modified RuO_2_ nanoparticles are designed to be loaded on hierarchical porous carbon shells (Fe_SA_‐RuO_2_/HPCS) based on EMSI criterion. It is revealed that the Ru‐O‐Fe_1_ structure is formed between the atomically dispersed Fe atoms and the surrounding Ru sites through electron interaction, and this structure could act as the ultra‐high activity driving force center of oxygen reduction/evolution reaction (ORR/OER). Specifically, the Ru‐O‐Fe_1_ structure enhances the reaction kinetics of ORR to a certain extent, and optimizes the morphology of discharge products by reducing the adsorption energy of catalyst for O_2_ and LiO_2_; while during the OER process, the Ru‐O‐Fe_1_ structure not only greatly enhances the reaction kinetics of OER, but also catalyzes the efficient decomposition of the discharge products Li_2_O_2_ by the favorable electron transfer between the active sites and the discharge products. Hence, LOBs based on FeSA‐RuO_2_/HPCS cathodes show an ultra‐low over‐potential, high discharge capacity and superior durability.

## Graphical abstract

Based on the electronic metal‐support interaction criterion, an atomically dispersed Fe‐modified RuO_2_ is designed as an efficient catalyst for Li‐O_2_ battery. The Ru‐O‐Fe_1_ structure formed in this catalyst changes the coordination environment of Ru sites and modulates the electronic structure, thus promoting the growth and decomposition of Li_2_O_2_ and significantly enhancing the kinetics of oxygen reduction reaction/oxygen evolution reaction reaction.

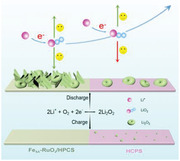

## 1 Introduction

Nowadays, growing of the market for electric vehicles and various electronic products has stimulated the development of efficient and sustainable energy conversion and storage technologies.^[^ ^1^, ^2^, ^3^ ^]^ In this context, the widely used lithium ion batteries gradually fail to meet the needs of people due to their unsatisfactory energy density (200–400 Wh kg^−1^), potential safety risks and unfriendly characteristics to the environment.^[^ ^3^, ^4^ ^]^ Recently developed rechargeable Li‐O_2_ batteries (LOBs) have attracted extensive attention and research due to their extremely high theoretical energy density (≈3500 Wh kg^−1^), and it even considered as an ideal power system for new electric vehicles.^[^ ^5^, ^6^ ^]^ In general, a typical LOB consists of a lithium metal anode, a separator, a porous O_2_ cathode, and an organic electrolyte with dissolved organic/inorganic lithium salts.^[^ ^6^ ^]^ It is generally believed that the high specific energy of LOBs comes from the electrochemical reaction between reaction gas (O_2_) and lithium ions on the cathode surface (2Li^+^ + 2e^−^ + O_2_ = Li_2_O_2_, *E^*θ*^* = 2.96 V vs Li^+^/Li).^[^ ^7^, ^8^ ^]^ As the main region of discharge–charge reaction, the pore structure, catalytic activity and durability of O_2_ cathodes can directly determine the electrochemical performance of LOBs to a large extent.^[^ ^6^, ^7^, ^8^, ^9^, ^10^ ^]^ In terms of the cathodes, however, there are some challenges that need to be addressed in order to make LOBs practical. Among them, the slow oxygen reduction reaction/oxygen evolution reaction (ORR/OER) kinetics, and the insulated and insoluble discharge products Li_2_O_2_, are the most intractable and pressing challenges.^[^ ^11^, ^12^, ^13^, ^14^ ^]^ Hence, cathodes with ultra‐high catalytic activity and durability can often significantly improve the electrochemical performance of LOBs.^[^ ^15^, ^16^, ^17^, ^18^ ^]^ Moreover, the ideal LOBs cathode materials should have high specific surface area and appropriate pore structure to provide enough channels and space for ion/gas transport and discharge products accumulation.^[^ ^19^, ^20^ ^]^ So the development of cathodes with high catalytic activity, durability and suitable pore structure is of great significance for the practical application of LOBs.

RuO_2_ catalysts with rutile structure exhibit excellent catalytic activity for LOBs and ability to regulate the growth pathway of discharge products, so RuO_2_ is recognized as the benchmark catalyst in LOBs and has been widely studied.^[^ ^21^, ^22^, ^23^ ^]^ But objectively speaking, the ORR/OER catalytic activity and durability of RuO_2_‐based catalysts still have room for improvement. Recently, some reports claim that the incorporation of transition metals can modulate the intrinsic electrocatalytic properties of metal oxides by improving its reactive electron surface distribution/coordination environment.^[^ ^24^, ^25^, ^26^ ^]^ This is mainly due to the interaction between the introduced component and the support material that regulates the electronic structure at the catalyst surface, and this phenomenon is known as the electronic metal‐support interaction (EMSI) or strong metal‐support interaction (SMSI).^[^ ^27^, ^28^, ^29^, ^30^ ^]^ Many reports have shown that catalysts based on EMSI/SMSI criterion designs tend to show increased catalytic activity/durability and powerful functionality.^[^ ^27^, ^30^ ^]^ For example, Xin et al. found that SMSI between Ru nanoparticles and MoO_3_ could optimize the electronic structure on the surface of Ru nanoparticles, thus enhancing the catalytic activity and selectivity of the catalyst for CO_2_ hydrogenation.^[^ ^28^ ^]^ Hou et al. demonstrated that W atoms deposited on the surface of PdO nanoparticles could form Pd‐O‐W_1_ structure, so as to optimize the oxygen activation pathway and reaction mechanism of methane combustion at low temperature.^[^ ^29^ ^]^ Using the electronic interaction between Mn atoms and RuO_2_, Zhou et al. identified the single atom Mn site and the modulated Ru site as excellent ORR and OER active sites respectively, which enabled the Zn‐air battery based on this catalyst to display extraordinary electrochemical performance.^[^ ^30^ ^]^ Although the catalysts designed based on EMSI/SMSI criterion have been successfully used in many fields, there are few reports on LOBs. Hence, it is significant to study the practicality and application potential of catalysts designed based on SMSI/EMSI criterion in LOBs field.

Hence, a novel strategy is proposed in this work, which can deposit atomically dispersed Fe atoms onto ultrafine RuO_2_ nanoparticles (NPs) and forming a Ru‐O‐Fe_1_ structure. The obtained catalyst is supported by hierarchical porous carbon shell (HPCS), and marked as Fe_SA_‐RuO_2_/HPCS. Experimental results not only confirm the atomic dispersion of Fe species on Fe_SA_‐RuO_2_ NPs, but also clearly analyze the interactions between the introduced Fe atoms and RuO_2_. These interactions including the formation of Ru‐O‐Fe_1_ structure, redistribution of the surface electronic structures, the elongation of Ru—O bond and the change of coordination environment of Ru atoms. Density functional theory (DFT) calculation is used to deeply understand the mechanism of Fe_SA_‐RuO_2_/HPCS cathode in LOBs. For the ORR process, compared with RuO_2_, the doping of Fe atom leads to a slight negative shift in the *d*‐band center of the Fe_SA_‐RuO_2_, which further results in its anti‐bonding orbital being lower than the Fermi level (*E*_f_), thus weakening the adsorption of O_2_ and LiO_2_ on Fe_SA_‐RuO_2_. The moderate adsorption energy of the Fe_SA_‐RuO_2_/HPCS cathode for O_2_ and LiO_2_ can optimize the growth pathway of discharge products, at the same time, the Ru‐O‐Fe_1_ structure can also improve the reaction kinetics of ORR. While during the OER process, the Ru‐O‐Fe_1_ structure can significantly improve the reaction kinetics of OER and reduce the resistance of O_2_ and LiO_2_ in the desorption process. In addition, there is a strong favorable electron transfer between the Ru‐O‐Fe_1_ structure and the discharge products Li_2_O_2_, these factors together promote the efficient decomposition of the discharge products during the charging process. Therefore, LOBs based on Fe_SA_‐RuO_2_/HPCS cathodes exhibit extraordinary electrochemical performances, including an excellent round‐trip efficiency, high discharge capacity and impressive cycle life (232 cycles). Additionally, Fe_SA_‐RuO_2_/HPCS cathodes also exhibit amazing durability during battery operation. This work not only reveals the beneficial effect of electron interaction between Fe atoms and RuO_2_ on ORR/OER, but also confirms the great potential of catalysts designed based on EMSI/SMSI criterion in LOBs.

## 2 Results and Discussion

**Figure 1:**
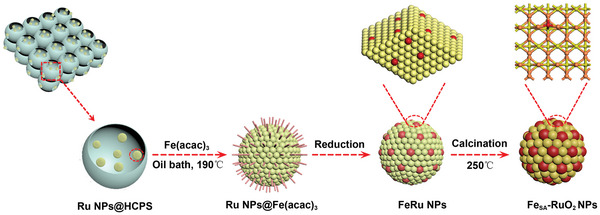
Schematic illustration of the synthesis process of the Fe_SA_‐RuO_2_/HPCS.

First, HPCS derived from sodium citrate is used as the substrate carbon material. According to our previous reports,^[^ ^31^, ^32^ ^]^ as the cathode of Li‐O_2_ or Li‐CO_2_ batteries, HPCS have advantages such as hollow spherical shell with thin shell, abundant pore and 3D cross‐linking structure, which can provide a large number of fast channels for gas/ions and have enough space to store discharge products. Subsequently, HPCS supported Ru NPs are prepared by liquid‐phase reduction method, and a small amount of Fe(acac)_3_ is added to deposit atomically dispersed Fe species on the surface of Ru NPs (FeRu NPs/HPCS). Finally, the FeRu NPs/HPCS sample is calcined in air for 30 min at 250 °C to obtain Fe_SA_‐RuO_2_/HPCS. The whole synthesis process is as observed in **Figure** **1**. By controlling the amount of Fe(acac)_3_, three Fe_SA_‐RuO_2_/HPCS samples (Fe_10_‐RuO_2_/HPCS, Fe_15_‐RuO_2_/HPCS, and Fe_20_‐RuO_2_/HPCS) with different Fe content are prepared. In addition, Fe_2_O_3_/HPCS and RuO_2_/HPCS samples are also synthesized for comparison. The surface morphology of these samples is identified by the scanning electron microscopy (SEM) technique (Figure S1, Supporting Information). All the samples show semi‐open spherical shell morphology with very thin shells (≈10 nm) and cross‐linking with each other, and the sizes of these spherical shells range from tens to hundreds of nanometers, which are typical morphological characteristics of HPCS.^[^ ^31^, ^32^, ^33^ ^]^ It is worth mentioning that the spherical shell surfaces on all samples are relatively smooth and no significant nanoparticles are observed, suggesting that the loaded nanoparticles should be very fine.^[^ ^25^ ^]^ To analyze NPs on these samples, they are observed by using transmission electron microscopy (TEM). TEM images confirm the semi‐open spherical shell and thin shell characteristics of HPCS (Figure S2a, Supporting Information), and observed dense distribution of small nanoparticles on the other samples (Figure S2b–e, Supporting Information). HRTEM images show that *d*‐spacings of about 0.256 and 0.169 nm are observed on both pristine RuO_2_ and three Fe_SA_‐RuO_2_ NPs samples with different Fe contents, which corresponding to the (101) and (211) planes of rutile RuO_2_,^[^ ^23^, ^24^, ^25^, ^26^ ^]^ respectively (Figure S3, Supporting Information), and no lattice fringes attributed to ferric oxide species are observed, which initially ruled out the possibility of small ferric oxide clusters or nanoparticles on these Fe_SA_‐RuO_2_ NPs. The selected‐area electron diffraction result of Fe_SA_‐RuO_2_/HPCS sample further confirm that RuO_2_ particles are rutile RuO_2_ (Figure S4, Supporting Information). Then the actual metal contents of these samples are determined by the inductively coupled plasma optical emission spectrometer (ICP‐OES), as listed in Table S1, Supporting Information. Among them, the content of Ru in RuO_2_/HPCS sample is 8.47 wt%, while the Ru contents of the three Fe_SA_‐RuO_2_/HPCS samples with different Fe content are similar to that of RuO_2_/HPCS sample, and the Fe contents of three Fe_SA_‐RuO_2_/HPCS samples are 0.14, 0.19, and 0.23 wt%, respectively.

The phases of the samples are identified by the XRD and Raman technique. The XRD pattern presents a broad humps peak at 25° and an inconspicuous peak locates at 43°, corresponding to the graphitic (002) and (100) planes,^[^ ^31^, ^32^, ^33^ ^]^ respectively, which indicates the amorphous state of HPCS (Figure S5, Supporting Information). No other diffraction peaks are observed in the remaining samples, which may be due to the poor crystallinity of HPCS and the fine NPs in these samples. The Raman spectra in Figure S6, Supporting Information, suggests that all samples show significant D (defect) band and G (graphite) band at about 1350 and 1585 cm^−1^, with an *I*_D_/*I*_G_ of about 0.95, confirming the excellent conductivity of HPCS. In addition, the weak Raman peaks attribute to Fe_2_O_3_ and rutile structured RuO_2_ appear in Fe_2_O_3_/HPCS,^[^ ^34^, ^35^ ^]^ and RuO_2_/HPCS as well as Fe_SA_‐RuO_2_/HPCS samples,^[^ ^25^, ^32^ ^]^ respectively.

**Figure 2:**
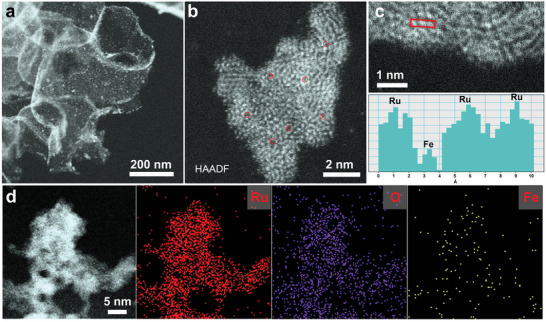
a–c) HAADF‐STEM images of Fe_SA_‐RuO_2_/HPCS at different magnification. d) STEM image and EDX elemental mappings of Fe_SA_‐RuO_2_/HPCS.

The high‐angle annular darkfield scanning transmission electron microscopic (HAADF‐STEM) and energy‐dispersive X‐ray (EDX) elemental mapping techniques are used to further analyze the microscopic morphology of the Fe_SA_‐RuO_2_/HPCS sample in detail. Among the three samples with different Fe contents, Fe_SA_‐RuO_2_/HPCS containing 0.19 wt% Fe species show the best catalytic activity for LOB (see below), so select it for HAADF‐STEM test. As observed in **Figure** **2**a and Figure S7a, Supporting Information, some irregularly shaped Fe_SA_‐RuO_2_ NPs are tightly and evenly loaded on the carbon shell. The particle size of Fe_SA_‐RuO_2_ NPs ranges from 2 to 8 nm, and some NPs are slightly agglomerated. The aberration‐corrected HAADF‐STEM (AC‐HAADF‐STEM) images show that some dark spots (highlighted in red circles) are dispersed on the surface of bright RuO_2_ NPs, which should be attributed to the atomically dispersed Fe atoms (Figure 2b,c). According to previous reports,^[^ ^11^, ^12^, ^15^ ^]^ the brightness of different species in the AC‐HAADF‐STEM image is proportional to the square of their relative atomic mass, whereas in the Fe_SA_‐RuO_2_/HPCS sample, the relative atomic mass of Fe is lower (compared with Ru) and therefore appears as dark spots.^[^ ^36^ ^]^ EDX elemental mapping is also performed to analyze the elemental distribution of the Fe_SA_‐RuO_2_/HPCS sample. As expected, the high‐magnification EDX analysis results also indicate that Fe species are atomically dispersed in the Fe_SA_‐RuO_2_/HPCS samples (Figure 2d). Notably, the uniform distribution of Fe_SA_‐RuO_2_ NPs on HPCS was further confirmed by the low‐magnification DEX element mapping images (Figure S7b, Supporting Information). In addition, the contents of Ru species and Fe atoms are 8.44 and 0.19 wt%, respectively, which are consistent with that of ICP‐OES (Table S1, Supporting Information). In conclusion, these characterization results preliminarily substantiate the existence of Fe species on the sample Fe_SA_‐RuO_2_/HPCS sample in atomically dispersed form.

To obtain the surface chemical states of different samples, these samples are analyzed by X‐ray photoelectron spectroscopy (XPS). The Ru 3p XPS spectra (Figure S8, Supporting Information) shows the presence of hydrated Ru oxide (465.9 eV) and Ru^4+^ (463.39 and 486.08 eV) for RuO_2_/HPCS.^[^ ^25^, ^32^ ^]^ And with the increase of Fe content in Fe_SA_‐RuO_2_/HPCS, the binding energy of each peak has more and more obvious negative shift, as shown in Figure S8b–d and Table S2, Supporting Information. These results indicate that the introduction of Fe atoms affects the electronic structure of RuO_2_, resulting in an increase in the electron density of the Ru sites in Fe_SA_‐RuO_2_. The O 1s XPS spectra (Figure S9a, Supporting Information) shows the oxygen‐containing functional groups peak at the 532.56 eV and surface adsorbed oxygen species peak at the 533.91 eV for HPCS.^[^ ^32^, ^33^ ^]^ In addition to the above two peaks, Fe_2_O_3_/HPCS, RuO_2_/HPCS and three Fe_SA_‐RuO_2_/HPCS observe two other peaks at 530.3 and 531.4 eV, which are attributed to lattice oxygen (M‐O) and hydroxyl group,^[^ ^24^, ^34^ ^]^ respectively, as shown in Figure S9b–f, Supporting Information. Fe 2p XPS spectra of Fe_2_O_3_/HPCS (Figure S10a, Supporting Information) shows two peaks at 712.12 and 725.65 eV, and a satellite peak at 717.47 eV, respectively, and all the peaks mentioned above are attributed to the Fe^3+^ species (Fe_2_O_3_).^[^ ^35^ ^]^ Remarkably, due to the low content of Fe, no Fe 2p related XPS signals in the three Fe_SA_‐RuO_2_/HPCS samples (Figure S10b, Supporting Information).

**Figure 3:**
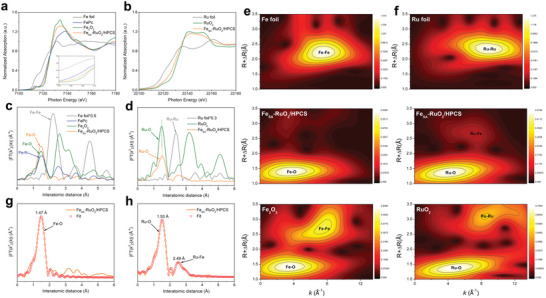
a) Fe K‐edge normalized XANES spectra. b) Ru K‐edge normalized XANES spectra. FT of the *k* ^2^‐weighted EXAFS spectra at c) Fe and d) Ru K‐edge. WT for the *k* ^2^‐weighted EXAFS signals at e) Fe and f) Ru K‐edge. The corresponding EXAFS fitting curves at g) Fe R space and h) Ru R space for Fe_SA_‐RuO_2_/HPCS.

To further understand the interactions of Fe species and RuO_2_ in the Fe_SA_‐RuO_2_/HPCS samples, X‐ray absorption spectroscopy (XAS) was carried out to probe the electronic state and coordination environment of Fe and Ru elements. As the Fe K‐edge X‐ray absorption near edge structure (XANES) profiles observed in **Figure** **3**a, the absorption edge position of Fe_SA_‐RuO_2_/HPCS situates between those of FePc and Fe_2_O_3_, confirming the presence of Fe species with valance between +2 and +3. While the Ru K‐edge XANES results show that the absorption edge position of Ru element in Fe_SA_‐RuO_2_/HPCS is slightly lower than that of RuO_2_ (Figure 3b). This indicates that the valence state of Ru species in Fe_SA_‐RuO_2_/HPCS is slightly lower than the +4 valence, which further confirms the conclusion of XPS. According to recent reports,^[^ ^30^, ^37^ ^]^ metal cations with lower valence states in metal oxides can induce unpaired d‐electrons and exhibit higher surface reactivity than metal cations with higher valence states. Hence, the lower valence states Ru cations means that Fe_SA_‐RuO_2_ NPs may show better catalytic activity than pure RuO_2_ NPs. Then, the local structural information around Fe/Ru was examined by the Fourier‐transformed (FT) *k* ^2^‐weighted extended X‐ray absorption fine structure (EXAFS) spectra in *R* space. The EXAFS spectra of Fe_SA_‐RuO_2_/HPCS (Figure 3c) exhibits a prominent peak around 1.47 Å at Fe K‐edge, which can be assigned to metal‐O scattering paths. Importantly, compared with Fe foil, no measurable metal‐metal scattering peak around 2.20 Å is detected for Fe_SA_‐RuO_2_/HPCS, which rule out the existence of small Fe nanoparticles or clusters. And another peak is observed at about 2.54 Å in the Fe K‐edge EXAFS spectra, while a weaker peak is also observed at the same position in the Ru K‐edge EXAFS spectra (Figure 3d). This scattering peak should be attributed to the Ru‐Fe bond (Ru‐O‐Fe_1_ structure in Fe_SA_‐RuO_2_ NPs), which is caused by the bonding of the doped Fe atom and the surrounding Ru atoms with the same O atom.^[^ ^26^, ^29^, ^38^ ^]^ In addition, it is also observed that the bond length of the Ru—O bond (1.53 Å) in Fe_SA_‐RuO_2_/HPCS is slightly stretched compared to commercial RuO_2_ (1.50 Å), which contributes to charge redistribution on Fe_SA_‐RuO_2_.^[^ ^29^, ^30^ ^]^

Wavelet transform (WT) analysis is further conducted to study the Fe and Ru K‐edge EXAFS oscillations of Fe_SA_‐RuO_2_/HPCS. Due to the WT contour plots with intensity maximums located at coordinates of (*k*, *R*) are closely related to the path length *R* and atomic number *Z*, WT‐EXAFS spectra could provide momentous clues for identifying coordination structures.^[^ ^39^ ^]^ As shown in Figure 3e, Fe_SA_‐RuO_2_/HPCS shows an intensity maximum at (3.7 Å^−1^, 1.3 Å), which is very close in both wave vector *k* and bond length *R* relative to Fe_2_O_3_ (3.6 Å^−1^, 1.3 Å), indicating that Fe species are mainly anchored to Fe_SA_‐RuO_2_ NPs by Fe‐O bond. Moreover, Fe_2_O_3_ also has an apparent maximum intensity attributed to the Fe—Fe bond at (7.9 Å^−1^, 2.7 Å), while Fe_SA_‐RuO_2_/HPCS does not, which further confirms the atomic dispersion of Fe species. And as observed in Figure 3f, Fe_SA_‐RuO_2_/HPCS shows an intensity maximum at (4.1 Å^−1^, 1.3 Å), but the abscissa value representing the path length *R* is obviously different from RuO_2_ (5.0 Å^−1^, 1.3 Å), which further confirmed that the introduction of Fe species changed the Ru‐O bond length. Note that, in RuO_2_ sample, a peak attributed to the Ru—Ru bond (Ru‐O‐Ru structure in RuO_2_) is shown at (8.6 Å^−1^, 3.1 Å), while in Fe_SA_‐RuO_2_/HPCS sample, this peak is missing, but a new peak appears at (7.1 Å^−1^, 2.7 Å), which should be attributed to the Ru‐Fe bond (Ru‐O‐Fe_1_ structure in Fe_SA_‐RuO_2_ NPs). The corresponding fitting curves of EXAFS spectra of Fe_SA_‐RuO_2_/HPCS in *R* space and *k* space are shown in Figure 3g,h and Figure S11, Supporting Information, to obtain the quantitative chemical configuration of Fe atoms and Ru species. For Fe atoms, the fitting result depicts that the coordination number is 5.4 ± 0.4, and with a mean bond distance of 1.95 Å (Table S3, Supporting Information). The fitting result depicts that the coordination number of Ru species is 5.2 ± 0.7 for Ru—O bond and 1.7 ± 0.3 for Ru—Fe bond (Table S3, Supporting Information). Besides, the mean Ru—O bond distance of Fe_SA_‐RuO_2_/HPCS sample is about 2.01 Å, which is longer than the mean Ru—O bond distance of RuO_2_ sample (1.98 Å). To sum up, the overall analysis of XPS and XAS results indicate that the introduced Fe species are atomically dispersed and formed a Ru‐O‐Fe_1_ structure on Fe_SA_‐RuO_2_ NPs. In addition, the introduction of Fe atoms increases the electron density of the Ru sites in RuO_2_, and slightly stretches the Ru—O bond.

To further understand the positions of doped Fe atoms in Fe_SA_‐RuO_2_ NPs, the formation energies of Fe atoms at different positions in RuO_2_ crystal structure are analyzed by DFT simulations. It is shown that, of all possible locations, Fe atoms are most likely to be anchored on Fe_SA_‐RuO_2_ NPs by replacing the Ru sites on the surface of RuO_2_ crystals due to thermodynamic advantages (Figure S12, Supporting Information). The DFT simulation results are in good agreement with those deduced by our material synthesis process (Figure 1). In addition, due to the formation energy is too high, the doped Fe atoms will not lead to the formation of a large number of oxygen vacancies in Fe_SA_‐RuO_2_ NPs (Figure S13, Supporting Information), which is consistent with the O 1s XPS spectra results of these materials.

**Figure 4:**
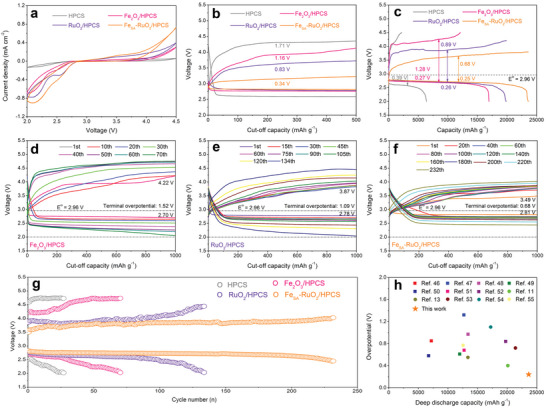
a) CV curves of different cathodes at a scan rate of 0.1 mV s^−1^ at a voltage window of 2.0–4.5 V. b) Discharge–charge curves of different cathodes at a curtailed capacity of 500 mAh g^−1^ at a current density of 100 mA g^−1^. c) Initial deep discharge–charge curves of different cathodes at a current density of 200 mA g^−1^. Discharge–charge profiles of d) Fe_2_O_3_/HPCS, e) RuO_2_/HPCS, and f) Fe_SA_‐RuO_2_/HPCS cathodes with different cycles at 200 mA g^−1^ and 1000 mAh g^−1^. g) Terminal discharge/charge voltage of LOBs based on different cathodes during cycle. h) Comparison of over‐potential and full discharge capacity between Fe_SA_‐RuO_2_/HPCS cathode and some RuO_2_‐based and single atom catalysts‐based LOBs (the numbers represent the serial numbers of references).

To evaluate the catalytic activity of the elaborately designed Fe_SA_‐RuO_2_/HPCS cathode toward LOBs, a series of electrochemical tests are conducted. **Figure** **4**a and Figure S14a, Supporting Information, exhibits the cyclic voltammograms (CV) profiles of the six samples under the scanning speed of 0.1 mV s^−1^ within electrochemical window of 2.0–4.5 V. Compared with HPCS, Fe_2_O_3_/HPCS, and RuO_2_/HPCS cathodes, the three Fe_SA_‐RuO_2_/HPCS cathodes with different Fe contents all showed higher ORR onset potential, lower OER onset potential and larger redox peak area. This indicates that the introduction of Fe atoms indeed improves the catalytic activity of Fe_SA_‐RuO_2_/HPCS cathode. In addition, CV results also suggest that for the three Fe_SA_‐RuO_2_ /HPCS cathodes, their catalytic activity to LOBs is in the order of Fe_15_‐RuO_2_/HPCS > Fe_10_‐RuO_2_/HPCS > Fe_20_‐RuO_2_/HPCS (the number represents the amount of iron precursor), which is also confirmed by the discharge–charge over‐potential in Figure S14b, Supporting Information. At 100 mA g^−1^ current density and 500 mAh g^−1^ cut‐off capacity, LOBs based on HPCS, Fe_2_O_3_/HPCS, and RuO_2_/HPCS cathodes show over‐potentials of 1.71, 1.16, and 0.83 V, respectively (Figure 4b). Surprisingly, the Fe_15_‐RuO_2_/HPCS cathode show over‐potential as low as 0.34 V, with terminal voltages of discharge and charge being 2.81 and 3.22 V, respectively. Moreover, Fe_10_‐RuO_2_/HPCS and Fe_20_‐RuO_2_/HPCS cathodes also show low over‐potential of 0.47 and 0.59 V, respectively (Figure S14b, Supporting Information). It is well known that in LOBs, discharge–charge over‐potential and CV test can directly reflect the catalytic activity of the catalyst,^[^ ^40^, ^41^ ^]^ so the above results jointly confirm that Fe_15_‐RuO_2_/HPCS cathode has the best catalytic activity for LOBs. As a result, Fe_15_‐RuO_2_/HPCS cathode is selected as the representative of Fe_SA_‐RuO_2_/HPCS cathode for other electrochemical tests.

Figure 4c shows the deep discharge–charge curves of different cathodes within a voltage window of 2.0 to 4.5 V at a current density of 200 mA g^−1^. It is obvious that the Fe_SA_‐RuO_2_/HPCS cathode exhibits a much higher capacity of 23 628 mAh g^−1^ in comparison with the RuO_2_/HPCS (19 891 mAh g^−1^), Fe_2_O_3_/HPCS (17 004 mAh g^−1^), and HPCS (6391 mAh g^−1^) cathodes. In addition, the Coulomb efficiencies of LOBs based on HPCS and Fe_2_O_3_/HPCS cathodes are 33.14% and 71.52%, respectively, while both RuO_2_/HPCS and Fe_SA_‐RuO_2_/HPCS cathodes achieve Coulomb efficiencies of 100% at a low charging potential. It is worth noting that the terminal charging potential of the RuO_2_/HPCS cathode is 4.20 V, while the terminal charging potential of the Fe_SA_‐RuO_2_/HPCS cathode is only 3.80 V. Even under higher current densities (300 mA g^−1^), Fe_SA_‐RuO_2_/HPCS retained a satisfactory depth discharge capacity (16 202 mAh g^−1^), while the discharge capacity of other cathodes decreased sharply (Figure S15a, Supporting Information). To exclude the possibility of battery self‐discharge and electrolyte decomposition, LOBs based on these cathodes were also assembled, and conducted deep discharge–charge tests under argon atmosphere. All cathodes display negligible discharge and charge capacity (Figure S15b, Supporting Information), suggesting that the capacity of LOBs is mainly derived from ORR/OER process.^[^ ^42^, ^43^ ^]^ Rate performance is also an important criterion for evaluating the catalytic activity of the cathodes to LOBs, so LOBs based on different cathodes was tested in the current density range of 100–500 mA g^−1^ (Figure S16, Supporting Information). Apparently, under all stages of current densities, the over‐potential from low to high is Fe_SA_‐RuO_2_/HPCS, RuO_2_/HPCS, Fe_2_O_3_/HPCS, and HPCS cathodes, which is consistent with the results of CV and deep discharge–charge tests.

Cycle stability is recognized as one of the most important criteria to measure the electrochemical performance of LOBs,^[^ ^17^, ^44^ ^]^ so the cyclic stability of LOBs based on different cathodes was tested at a current density of 200 mA g^−1^ and cut‐off capacity of 1000 mAh g^−1^. The experimental results show that before the terminal discharge potential decreases to 2.0 V, LOBs based on HPCS, Fe_2_O_3_/HPCS, and RuO_2_/HPCS cathodes can cycle 27, 70, and 134 cycles, respectively (Figure 4d,e and Figure S17, Supporting Information). And their terminal over‐potential of the first‐cycle is 2.08, 1.52, and 1.09 V, respectively. In contrast, the LOB based on Fe_SA_‐RuO_2_/HPCS cathode exhibit a much longer cycle life (232 cycles) and the first‐cycle terminal over‐potential is only 0.68 V, as shown in Figure 4f. The discharge/charge terminal potentials of LOBs based on different cathodes were also collected, which can visually demonstrate the difference of catalytic activity of different cathodes through the change of voltage platform (Figure 4g). It can be observed that Fe_SA_‐RuO_2_/HPCS cathode always show highest discharge potential and lowest charge potential than other cathodes in the same cycle period. Even at the 232th cycle, the terminal charge potential is only about 4.0 V. The impressive cyclic stability not only further confirms the excellent catalytic activity of Fe_SA_‐RuO_2_/HPCS cathode for LOBs, but also indicates its outstanding durability. To further highlight the catalytic activity and durability of Fe_SA_‐RuO_2_/HPCS cathode, the electrochemical performances of LOBs based on Fe_SA_‐RuO_2_/HPCS cathode were compared with some reported RuO_2_‐based and single atom catalysts‐based LOBs,^[^ ^11^, ^13^, ^45^, ^46^, ^47^, ^48^, ^49^, ^50^, ^51^, ^52^, ^53^, ^54^ ^]^ as shown in Figure 4h and Table S4, Supporting Information. It is obvious that LOBs based on Fe_SA_‐RuO_2_/HPCS cathode show electrochemical performances that far exceed those previously reported.

**Figure 5:**
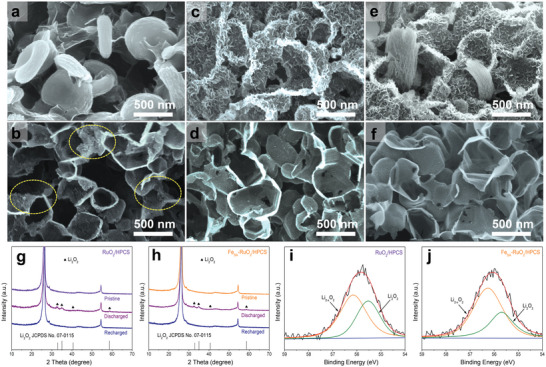
SEM images of a) HPCS, c) RuO_2_/HPCS, and e) Fe_SA_‐RuO_2_/HPCS cathodes after discharged. SEM images of b) HPCS, d) RuO_2_/HPCS, and f) Fe_SA_‐RuO_2_/HPCS cathodes after recharged. XRD patterns of pristine, discharged, and recharged g) RuO_2_/HPCS and h) Fe_SA_‐RuO_2_/HPCS cathodes. High‐resolution Li 1s XPS spectra of i) RuO_2_/HPCS and j) Fe_SA_‐RuO_2_/HPCS cathodes after discharged.

In order to achieve in‐depth unveiling of the working principles ORR/OER process on these cathodes, it is of great significant to observe the microstructure evolution of the discharged/recharged cathodes. Hence, SEM technique was used to observe the cathodes at various stages (pristine, discharged, and recharged) under the same conditions, as shown in **Figure** **5**a–f and Figure S18, Supporting Information. It can be observed that the pristine HPCS, Fe_2_O_3_/HPCS, RuO_2_/HPCS, and Fe_SA_‐RuO_2_/HPCS cathodes all present similar cross‐linked semi‐open carbon shell morphology (Figure S18a–d, Supporting Information). After discharged, disk‐shaped discharge products appeared on the surface of HPCS cathode (Figure 5a). According to related reports,^[^ ^3^, ^7^ ^]^ the formation of this morphology is mainly caused by the solvation path growth of discharge products. And after recharged, some residual small particles can be observed on the cathode surface of HPCS, as shown in Figure 5b. This may be due to the unsatisfactory catalytic activity of HPCS, which cannot catalyze the complete decomposition of the discharge products. The discharge products on Fe_2_O_3_/HPCS and RuO_2_/HPCS cathodes show a sheet‐shaped growing along the shell wall of the carbon shell (Figure 5c and Figure S19a, Supporting Information), indicating that their discharge products formed by surface‐adsorption growth pathway.^[^ ^19^, ^47^, ^52^ ^]^ In contrast, the size of sheet‐shaped discharge products on Fe_2_O_3_/HPCS cathodes are slightly larger than those on RuO_2_/HPCS cathodes, which may be due to the different adsorption energy of Fe_2_O_3_ and RuO_2_ NPs to intermediate.^[^ ^11^, ^52^ ^]^ After recharged, the surface morphology of Fe_2_O_3_/HPCS and RuO_2_/HPCS cathodes basically returned to their pristine morphology (Figure 5d and Figure S19b, Supporting Information), indicating their better ability to catalytically decompose the discharge products than the HPCS cathodes. Quite surprisingly, after discharged, two different patterns of discharge products that are sheet‐ and disk‐shaped were observed on the surface of Fe_SA_‐RuO_2_/HPCS cathode (Figure 5e). According to relevant reports,^[^ ^7^, ^8^, ^9^, ^10^ ^]^ the reason for this phenomenon may be that the active sites have moderate adsorption energy for the reaction intermediates (see below). The discharge products on Fe_SA_‐RuO_2_/HPCS cathode coexist in two forms, making the space inside the carbon shell more fully utilized, which perfectly explains why the deep discharge capacity of Fe_SA_‐RuO_2_/HPCS cathode is much larger (Figure 4c). In the OER process, the densely distributed Fe_SA_‐RuO_2_ NPs with ultra‐high catalytic activity are closely combined with the discharge products, which greatly reduces the charging potential and avoids the residue of the discharge products. Undoubtedly, the surface morphology of the cathode completely recovered after the charging process due to the superior catalytic activity of Fe_SA_‐RuO_2_/HPCS (Figure 5f).

Then, XRD and XPS measurements were used to further investigate the composition of the discharge products on different cathodes. As shown in Figure 5g,h and Figure S20, Supporting Information, after discharged, new diffraction peaks consistent with the standard diffraction peak of Li_2_O_2_ (JCPDS 07–0115) appeared on all the cathodes, and the corresponding diffraction peaks disappeared after recharged. It is noteworthy that although the four cathodes discharge at the same cut‐off capacity, their diffraction peaks intensity of discharge products shows significant differences. Specifically, the diffraction peaks intensity of discharge products Li_2_O_2_ is in the order of HPCS > Fe_2_O_3_/HPCS > RuO_2_/HPCS > Fe_SA_‐RuO_2_/HPCS, which indicates that the discharge products on the Fe_SA_‐RuO_2_/HPCS cathode has the worst crystallinity.^[^ ^20^ ^]^ The results of the high‐resolution Li 1s XPS spectra of the different cathodes after discharge also show that the proportion order of the amorphous discharge products (Li_2_O_2‐_ *_x_*) is Fe_SA_‐RuO_2_/HPCS > RuO_2_/HPCS > Fe_2_O_3_/HPCS > HPCS (Figure 5i,j and Figure S21, Supporting Information).^[^ ^20^ ^]^ This may be because in this work, the catalytic activity of different cathodes is very different, leading to different ORR kinetic speed.^[^ ^6^, ^7^, ^8^, ^9^ ^]^ In addition, the adsorption energy of catalyst for LiO_2_ and/or Li_2_O_2_ is also an important factor for the crystallization of discharge products.^[^ ^10^, ^11^, ^12^, ^13^ ^]^ Previous reports have shown that amorphous discharge products (Li_2_O_2‐_ *_x_*) are more likely to be catalyzed during OER process than that of well‐crystallinity discharge products (Li_2_O_2_).^[^ ^55^ ^]^ Corresponding electrochemical impedance tests confirmed that the impedance values of all cathodes increased significantly after discharge and returned to near original values after charging (except for HPCS cathodes), as observed in Figure S22, Supporting Information. Moreover, in situ differential electrochemical mass spectrometry was used to further analyze the reversibility of LOBs based on HPCS, Fe_2_O_3_/HPCS, RuO_2_/HPCS, and Fe_SA_‐RuO_2_/HPCS cathodes (Figure S23, Supporting Information). According to the theoretical equation of LOBs (Li_2_O_2_ → 2Li^+^ + 2e^−^ + O_2_), the ratio of the transferred electrons to the gas in the OER process should be 2:1, and O_2_ should be detected as the only gaseous product.^[^ ^7^, ^8^, ^9^, ^10^ ^]^ However, the obvious CO_2_ gas product were detected in LOBs based on HPCS and Fe_2_O_3_/HPCS cathodes during OER, implying that more unsatisfied reactions are occurring. In stark contrast, LOBs based on RuO_2_/HPCS and Fe_SA_‐RuO_2_/HPCS cathodes, only O_2_ is detected and more O_2_ is released on the Fe_SA_‐RuO_2_/HPCS cathode, indicating that it has the best catalytic activity for LOBs among these cathodes.

An ideal LOBs cathode should not only have desired catalytic activity, but also excellent durability. Therefore, SEM techniques were used to characterize different cathodes after a certain cycles number to observe the decomposition of their discharge products. After 10 cycles, a small amount of undecomposed discharge products could be observed on the surface of the HPCS cathode (Figure S24a, Supporting Information). When the battery continued to run until it failed, it was found that the film accumulated by the residual discharge products had covered most of the surface of the carbon shell (Figure S24b, Supporting Information), which may be due to the limited catalytic activity of the HPCS cathode itself and the unsatisfactory contact interface between HPCS and the discharge products.^[^ ^8^, ^10^ ^]^ As for Fe_2_O_3_/HPCS cathode, after 10 cycles, some fine granular discharge products can be observed on its surface (Figure S25a, Supporting Information). As the cycle goes on, the residual discharge products gradually accumulating until the battery fails (Figure S25b–d, Supporting Information). The gradual accumulation of discharge products and/or parasitic products indicate that the insufficient catalytic activity and durability of Fe_2_O_3_/HPCS cathodes. As shown in Figure S26a–c, Supporting Information, the RuO_2_/HPCS cathode can basically maintain its pristine morphology even after 90 cycles. However, a small amount of residual discharge products appeared on the surface of RuO_2_/HPCS cathode after battery failure (Figure S26d, Supporting Information). The outstanding durability of RuO_2_/HPCS cathode is consistent with previous reports of RuO_2_‐based catalysts in LOBs.^[^ ^45^, ^51^ ^]^ And as expected, the Fe_SA_‐RuO_2_/HPCS cathode with the best catalytic activity exhibited extraordinary durability. After 60, 120, and 180 cycles, the morphology of Fe_SA_‐RuO_2_/HPCS cathode is almost unchanged, and even after battery failure (232 cycles), only trace discharge products residues were observed on its surface (Figure S24, Supporting Information). Consider the superior catalytic activity and durability of the Fe_SA_‐RuO_2_/HPCS, the failure of the LOB based on Fe_SA_‐RuO_2_/HPCS cathode may be due to electrolyte volatilization and lithium anode degradation during battery operation (Figure S27, Supporting Information).^[^ ^6^, ^7^, ^8^, ^9^ ^]^ Remarkably, even after 60 cycles, atomic‐disperse black spots (representing Fe atoms) were still observed on the Fe_SA_‐RuO_2_/HPCS cathode, further highlighting the excellent durability of this catalyst (Figure S28, Supporting Information). Based on this perspective, a new LOB was assembled using the Fe_SA_‐RuO_2_/HPCS cathode obtained from the failed LOB, and this battery was found to be able to operate stably for more than 100 cycles (Figure S29, Supporting Information). In conclusion, that the elaborately designed Fe_SA_‐RuO_2_/HPCS cathode possess a superior catalytic activity as well as excellent durability.

**Figure 6:**
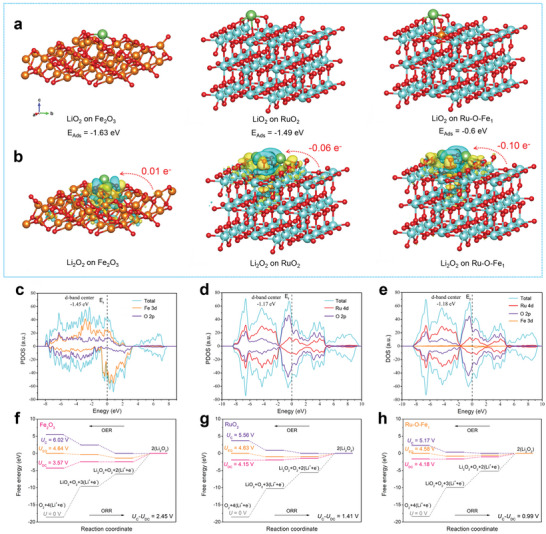
a) Adsorption energy of reaction intermediate LiO_2_ on Fe_2_O_3_, RuO_2_, and Ru‐O‐Fe_1_ active sites. b) Charge density differences for Li_2_O_2_* adsorption states and corresponding charge transfer on the Fe_2_O_3_, RuO_2_, and Ru‐O‐Fe_1_ active sites. The total density of states (DOS) and partial density of states (PDOS) of c) Fe_2_O_3_, d) RuO_2_, and e) Ru‐O‐Fe_1_ models. Calculated free energy diagrams for the discharge–charge reactions on the active sites of f) Fe_2_O_3_, g) RuO_2_, and h) Ru‐O‐Fe_1_.

DFT calculation was performed to deeply explore the source of the superior catalytic activity of Fe_SA_‐RuO_2_/HPCS cathode. Since the physical and chemical properties of cathode materials (HPCS) used in this work were basically consistent with previous work,^[^ ^31^, ^32^ ^]^ and the electrochemical properties and discharge products of LOBs based HPCS cathodes are also very close to our previous work,^[^ ^32^ ^]^ only catalytic components loaded on HPCS were considered. Hence, to simulate the active sites of Fe_2_O_3_/HPCS, RuO_2_/HPCS and Fe_SA_‐RuO_2_ /HPCS cathodes in LOBs, three models (Fe_2_O_3_, RuO_2_, and Ru‐O‐Fe_1_) were established. By comparing the adsorption energy (Δ*E*_Ads_) of the reaction gas O_2_ and the reaction intermediate LiO_2_ at different catalytic active sites, the reason for the difference in the morphology of the discharge products at different cathodes was analyzed. **Figure** **6**a and Figure S30, Supporting Information, reveal the optimized adsorption construction between the three active sites and reactants (O_2_ and LiO_2_). It was found that the adsorption energy of Fe_2_O_3_, RuO_2_, and Ru‐O‐Fe_1_ for the reaction gas O_2_ are −1.18, −1.49, and −0.99 eV, respectively, while their adsorption energies for reaction intermediate LiO_2_ are −1.63, −1.49, and −0.60 eV, respectively. In LOBs field, the adsorption energy of catalysts for reactants (especially for reaction intermediate) has a great influence on the growth pathway of discharge products.^[^ ^11^, ^12^, ^13^, ^14^ ^]^ For active sites Fe_2_O_3_ and RuO_2_, both of them have high adsorption energy for O_2_ and LiO_2_, so discharge products of Fe_2_O_3_/HPCS and RuO_2_/HPCS cathodes tend to form sheet‐like Li_2_O_2_ through the surface‐adsorption growth pathway (Figure 5c and Figure S19a, Supporting Information).^[^ ^19^, ^20^, ^52^ ^]^ And due to the slightly different adsorption energies of Fe_2_O_3_ and RuO_2_ sites to the reaction intermediate LiO_2_, their discharge products are slightly different in size. As for active site Ru‐O‐Fe_1_, although its adsorption energy for O_2_ and LiO_2_ is weak, considering that the Ru‐O‐Fe_1_ structure occupies only a part of Fe_SA_‐RuO_2_ NPs, the discharge product Li_2_O_2_ on Fe_SA_‐RuO_2_/HPCS cathode obtained two different morphologies by dual growth pathway.^[^ ^56^, ^57^ ^]^ During the OER process, the desorption of O_2_ and LiO_2_ on the active sites Fe_2_O_3_ and RuO_2_ is relatively unfavorable due to the large adsorption energy, resulting in their higher charging potential. Conversely, the weak adsorption energy favors the desorption of O_2_ and LiO_2_ and on the active site Ru‐O‐Fe_1_, thereby reducing the resistance experienced by the Fe_SA_‐RuO_2_/HPCS cathode during charging.^[^ ^13^ ^]^

It has been reported that in many electrocatalytic reaction systems,^[^ ^52^, ^58^, ^59^ ^]^ electron transfer between the active sites and the reactants is considered to be the key factor affecting the reaction process. Hence, the corresponding charge density difference is further employed to investigate the charge distributions on the three models, and the charge transfer between them and the discharge products Li_2_O_2_* (adsorption state), as observed in Figure 6b. The charge depletion (i.e., a loss of electron density) and accumulation (i.e., a gain of electron density) in these models are colored in cyan and yellow, respectively. Obviously, compared with the slight electron transfer between Fe_2_O_3_ site and Li_2_O_2_*, the electron transfer between RuO_2_ and Ru‐O‐Fe_1_ sites and Li_2_O_2_* is much stronger, and there is a clear cyan region (electron density loss) around Li_2_O_2_*. Specifically, for the adsorbed Li_2_O_2_*, Fe_2_O_3_ tends to transfer a small number of electrons (0.01 e) to Li_2_O_2_*, which is undoubtedly not conducive to the conversion process of Li_2_O_2_*. While adsorbed Li_2_O_2_* tends to transfer its electrons to the active sites RuO_2_ and Ru‐O‐Fe_1_, and the number of electron transfer between Ru‐O‐Fe_1_ and Li_2_O_2_* (0.10 e) is larger than that between RuO_2_ and Li_2_O_2_* (0.06 e). The increased electron transfer indicates that during charging, adsorbed Li_2_O_2_* can transfer its electrons to the active site of Ru‐O‐Fe_1_ more efficiently and promote the conversion process from Li_2_O_2_* to LiO_2_*, thus greatly enhancing the reaction kinetics on the active site Ru‐O‐Fe_1_.^[^ ^59^ ^]^ We also calculated the charge transfer between the active sites and the reaction intermediate LiO_2_* (adsorption state), as shown in Figure S31, Supporting Information. The calculated results show that the charge transfer between LiO_2_* and the active sites Fe_2_O_3_, RuO_2_, and Ru‐O‐Fe_1_ is −0.64, −0.50, and −0.45 eV, respectively. Although these active sites tends to transfer electrons to LiO_2_*, the charge transfer between Ru‐O‐Fe_1_ and LiO_2_* in the three active site models is the smallest, which further supports the best catalytic activity of Fe_SA_‐RuO_2_/HPCS cathode.

To reveal the physical origin of the difference in catalytic activity between RuO_2_ and Ru‐O‐Fe_1_ sites, the spin‐resolved density of states (DOS) of Fe_2_O_3_, RuO_2_, and Fe_SA_‐RuO_2_ are calculated (Figure 6c–e). It is found that the *d*‐band center of Fe_SA_‐RuO_2_ has a slight negative shift compared with that of RuO_2_. This will undoubtedly pull down the anti‐bonding orbital of the molecule, causing the anti‐bonding orbital to be occupied by more electrons, further causing the anti‐bonding orbital of the molecule to be lower than the Fermi level (*E*_F_), which is not conducive to its adsorption.^[^ ^13^, ^30^ ^]^ Furthermore, according to the partial density of states, the introduction of Fe atoms regulated the DOS of RuO_2_, so Fe atoms significantly contributed to the valence band and conduction band of the Fe_SA_‐RuO_2_ system.

Figure 6f–h give the calculated free energy pathways of discharge/charge processes based on nucleation/decomposition of (Li_2_O_2_)_2_ clusters at different over‐potentials for the three models. Then, the theoretical over‐potential obtained from gibbs free energy was studied and their catalytic activity was further analyzed. Here, *U*_DC_, *U*_C_, and *U*_EQ_ represent discharge voltage, charge voltage and equilibrium potential, respectively. Discharge (ORR) and charge (OER) over‐potential are defined as Δ_ORR_ = *U*_EQ_ − *U*_DC_ and Δ_OER_ = *U*_C_ − *U*_EQ_, respectively. While the overall over‐potential is defined as Δ_O_ = *U*_C_ − *U*_DC_.^[^ ^11^, ^52^, ^58^ ^]^ For active site Fe_2_O_3_, the calculated Δ_ORR_/Δ_OER_ value is 1.07/1.38 V (Figure 6f), while the corresponding Δ_ORR_/Δ_OER_ values for active sites RuO_2_ (Figure 6g) and Ru‐O‐Fe_1_ (Figure 6h) are 0.93/0.48 V and 0.58/0.40 V, respectively. The theoretical over‐potential Δ_O_ of active sites Fe_2_O_3_, RuO_2_, and Ru‐O‐Fe_1_ were 2.45, 1.41, and 0.99 V, respectively. Such calculation results are consistent with the experimental results in Figure 3, which also further confirms the best catalytic activity of Fe_SA_‐RuO_2_/HPCS cathode for LOBs. To sum up, both experimental results and theoretical calculations indicate that the Ru‐O‐Fe_1_ structure on the surface of Fe_SA_‐RuO_2_ NPs is the decisive factor for the Fe_SA_‐RuO_2_/HPCS cathode to exhibit extraordinary catalytic activity for LOBs.

**Figure 7:**
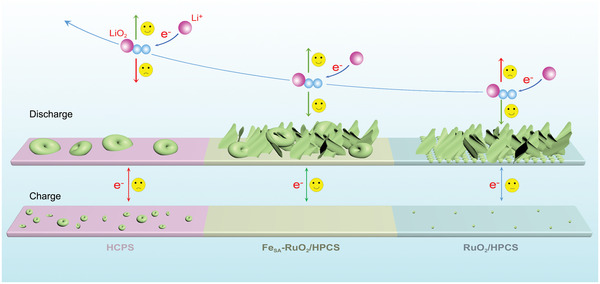
Schematic illustrations of the working mechanism for the HPCS, RuO_2_/HPCS, and Fe_SA_‐RuO_2_/HPCS cathodes.

Based on the DFT calculation and experimental results, we elucidated the rational way of the formation and decomposition of the discharge products on different cathodes, and the corresponding schematic diagrams are demonstrated in **Figure** **7** and Figure S32, Supporting Information. First, for HPCS cathode, because HPCS lack adsorption active sites for reactants,^[^ ^11^, ^12^, ^13^, ^14^ ^]^ Li_2_O_2_ forms disk‐like discharge products on its surface through solvation growth pathway (Figure 5a).^[^ ^6^ ^]^ During the subsequent charging process, due to the unsatisfactory catalytic activity of HPCS, and poor contact between the large size disk‐shaped discharge products and the carbon shell, incomplete decomposition of discharge products and high charging potential were resulted (Figure 5b). Second, for Fe_2_O_3_/HPCS and RuO_2_/HPCS cathodes, due to the strong adsorption energies of Fe_2_O_3_ and RuO_2_ for O_2_ and LiO_2_,^[^ ^6^, ^10^ ^]^ their discharge products all form sheet‐shaped Li_2_O_2_ through the surface‐adsorption growth pathway (Figure 5c and Figure S16e, Supporting Information). It is worth mentioning that the size difference of their discharge products is caused by the difference in their adsorption capacities for LiO_2_. During the charging process, since the OER catalytic activity of RuO_2_ is much better than that of Fe_2_O_3_, the RuO_2_/HPCS cathode has a lower charging potential and less residual discharge products during cycling. Last, for the Fe_SA_‐RuO_2_/HPCS cathode, the discharge products on the Fe_SA_‐RuO_2_/HPCS cathode grow through a dual growth pathway due to the existence of the Ru‐O‐Fe_1_ structure weakening the adsorption capacity of Fe_SA_‐RuO_2_ NPs for O_2_ and LiO_2_, two discharge products with different morphologies are formed (Figure 5e).^[^ ^56^, ^57^ ^]^ The coexistence of two different morphology of Li_2_O_2_ can make better use of the semi‐open space of the carbon shell, thereby enhancing the discharge capacity of Fe_SA_‐RuO_2_/HPCS cathodes. Accordingly, due to the superior catalytic activity of the Fe_SA_‐RuO_2_ NPs, the discharge products can be decomposed at an ultra‐low charging potential, while avoiding the residue of the discharge products (Figure 5f and Figure S27, Supporting Information).

## 3 Conclusion

In summary, RuO_2_ nanoparticles with atomically dispersed Fe atoms were designed based on EMSI criteria, and used it as efficient and durable catalyst for LOBs. The LOBs constructed based on this catalyst displayed extraordinary electrochemical properties, including an ultra‐low over‐potential (0.34 V), high discharge capacity (23 628 mAh g^−1^) and impressive cycle life (232 cycles). The potential factors of the extraordinary catalytic activity and durability for Fe_SA_‐RuO_2_/HPCS cathode are generalized as follows. First, the atom‐dispersed Fe atoms form Ru‐O‐Fe_1_ structure through electron interaction with RuO_2_, and improve the electron distribution of the original RuO_2_. Second, Ru‐O‐Fe_1_ sites optimize the morphology of discharge products and accelerate the decomposition of the discharge products by modulating the adsorption energy of the reactants (O_2_ and LiO_2_). Third, favorable electron transfer between the Ru‐O‐Fe_1_ sites and the discharge products can promote the decomposition of Li_2_O_2_. Last, the Ru‐O‐Fe_1_ sites as the powerful driving force center can significantly enhance the ORR/OER reaction kinetics of LOBs. This work not only confirms the feasibility of the catalysts designed based on EMSI criteria in LOBs field, but also provides some reference value for the design of LOBs catalysts with extraordinary catalytic activity and durability.

## Conflict of Interest

The authors declare no conflict of interest.

## Supporting information

Supporting Information

## Data Availability

The data that support the findings of this study are available from the corresponding author upon reasonable request.
