# Supplementary material for: Engineering the Electronic Interaction between Atomically Dispersed Fe and RuO2 Attaining High Catalytic Activity and Durability Catalyst for Li‐O2 Battery
Source: Adv Sci (Weinh). 2023 Jan 22;10(9):2205975. doi: 10.1002/advs.202205975 (PMC10037969; doi:10.1002/advs.202205975)
Supplement: Supplementary file 1 — Supporting Information [file ADVS-10-2205975-s001.pdf]

Supporting Information

Engineering the Electronic Interaction Between Atomically Dispersed Fe and RuO_2 Attaining High Catalytic Activity and Durability Catalyst for Li-O_2 Battery

Zheng Lian[a,b], Youcai Lu[a,], Shaoze Zhao[a], Zhongjun Li[a], Qingchao Liu[a,]**

[a]Green Catalysis Center, and College of Chemistry, Zhengzhou University, Zhengzhou, 450001, PR China.

[b]State Key Laboratory of Pulp and Paper Engineering, South China University of Technology, Guangzhou 510641, PR China

***Corresponding authors**

Email: yclu@zzu.edu.cn; qcliu@zzu.edu.cn.

1. Experimental section

Chemicals and Materials. sodium citrate dihydrate ($Na_3C_6H_5O_7·2H_2O$, Aladdin, ≥99%), ruthenium chloride hydrate ($RuCl_3·xH_2O$, Aladdin, 99.95%), sodium bromide (NaBr, $C_4H_6N_2$, Aladdin, 99%), D-glucose ($C_6H_{12}O_6$, Energy Chemical, 99%), polyvinylpyrrolidone (PVP, Aladdin, K16-18), ethylene glycol ($C_2H_6O_2$, Energy Chemical, 99.5%), Iron(III) acetylacetonate ($Fe(acac)_3$, Aladdin, 98%), tetraethylene glycol dimethyl ether (TEGDME, Aladdin, ≥99.5%), lithium trifluoromethanesulfonate (CF_3SO_3Li, Aladdin, ≥99.5), Deionized water.

Electrocatalyst preparation. Preparation of HPCS: The synthesis method of HPCS was improved on the basis of previous report.[1-3] Weigh 2g sodium citrate ($Na_3C_6H_5O_7·2H_2O$) and grind it for 15 minutes, and then transfer it to an oven at 150°C for 24 hours. The obtained powder was then ground again for 20 minutes. The carefully ground powder was placed in a ceramic boat and transferred into a tube furnace. The temperature was heated to 800°C at a rate of 5°C min^{-1} under N_2 atmosphere for 2 hours and then cooled naturally to room temperature. The collected black powder was cleaned to neutral with deionized water and dried overnight in an oven at 60°C to obtain HPCS.

Preparation of FeRu NPs/HPCS: 15 mg HPCS, 20 mg $RuCl_3·xH_2O$, 50 mg NaBr, 60 mg D-glucose and 100 mg PVP were placed in the flask, followed by 20 ml of ethylene glycol (EG) and sonication for 30 min. Next, the magnetic stirrer was placed in a flask and stirred under an oil bath at 190°C for 20 minutes. Followed by the addition of 10/15/20 mg $Fe(acac)_3$ and continued stirring for 220 minutes. After

cooling to room temperature, the black sample powder was collected by centrifugation, and the sample was washed several times with ethanol and deionized water, and dried overnight in an oven at 60°C. Note that, the preparation process of Ru NPs/HPCS is similar to that of FeRu NPs/HPCS, except that no $Fe(acac)_3$ was added.

Preparation of Fe_{SA}-RuO_2/HPCS: The obtained FeRu NPs/HPCS samples were placed in a crucible bowl and heated to 250°C for 30 minutes at a heating rate of 5°C/min in muffle furnace to obtain Fe_{SA}-RuO_2/HPCS.

2. Materials Characterizations

The morphology of samples was characterized by transmission electron microscopy (JEM-2100 TEM) and scanning electron microscopy (Zeiss SIGMA 500 SEM). XPS was performed on a Thermo escalab 250Xi instrument with Al Ka radiation. The crystal structures of samples were characterized by an X-ray diffraction (XRD, Cu-kα radiation). Raman spectra were performed on Renishaw with a 532 nm laser. Atomic-level high-angle annular dark-field scanning TEM (HAADF-STEM) images were recorded from a probe corrected TEM (JEM-ARM200F) working at 200 kV, coupled with double probe spherical aberration correctors. The X-ray absorption fine structure spectra (Fe and Ru K-edge) were collected at BL14W beamline in Shanghai Synchrotron Radiation Facility (SSRF). The storage rings of SSRF were operated at 3.5 GeV with a stable current of 200 mA. Using Si(111) double-crystal monochromator, the data collection were carried out in fluorescence mode using Lytle detector. All spectra were collected in ambient conditions. The XAFS results were

fitted via the IFEFFIT software. DEMS was performed on *i*-DEMS 100. The content of the Fe and Ru was measured by inductively coupled plasma-optical emission spectrometer (ICP-OES, Thermo Fisher 6500).

3. Assembly and testing of LOBs. The prepared cathode materials (HPCS, Fe_2O_3/HPCS, RuO_2/HPCS and Fe_{SA}-RuO_2/HPCS) was mixed with PVDF at a weight ratio of 95:5, and an appropriate amount of NMP was added and ground carefully to form a uniform slurry. The cathodes of LOBs can be obtained by evenly spreading the paste on the pre-cut circular carbon paper and drying it overnight in an oven at 60°C. Note that, the cathode active materials loading was ~0.3 mg cm^{-2}, the discharge/charge specific capacity of cathodes loaded with catalysts is calculated based on the mass of the overall active materials (HPCS and nanoparticle catalyst). The battery model used is the 2025-type coin battery. All of the batteries were assembled in an Ar gas-filled glove box with O_2 and H_2O contents less than 0.1 ppm. And the LOBs consist of a lithium foil anode (10 mm diameter, 0.5 mm thick), a glass fibre separator (19 mm diameter, GF/D, Whatman), a cathode and an electrolyte containing 1 M $LiSO_3CF_3$ in TEGDME. Then transfer the prepared battery to a sealed glass bottle filled with O_2 (99.999%), and perform galvanostatic charge-discharge cycles on Neware battery testing system (CT-4008Tn-5V10mA-HWX, Shenzhen, China). Cyclic voltammetry (CV) was test at a scan rate of 0.1 mV s^{-1} within a voltage range of 2.0-4.5 V on an electrochemical workstation (CHI 660E). All the electrochemical data were recorded at room temperature.

4. Computational details. *Computational method*: Vienna Ab Initio Package (VASP)[4-6] was employed to perform all the spin-polarized density functional theory (DFT) calculations within the generalized gradient approximation (GGA) using the Perdew-Burke-Ernzerhof (PBE) formulation.[7] The Hubbard U (DFT+U) corrections of transition metals (4.0/Ru, 3.4/Fe) were considered in the calculations.[8] The projected augmented wave (PAW) potentials were chosen to describe the ionic cores and take valence electrons into account using a plane wave basis set with a kinetic energy cutoff of 450 eV.[9] Partial occupancies of the Kohn-Sham orbitals were allowed using the Gaussian smearing method and a width of 0.05 eV. The electronic energy was considered self-consistent when the energy change was smaller than 10^{-5} eV. The maximum Hellmann-Feynman force for each ionic optimization step is 0.05 eV/Å, as well as the optimization of equilibrium lattice constants.

Model building: The configurations of catalysts are established, named RuO_2 (110) and Fe-doped. A p(4x2) unit cell was chosen for both catalysts, and six layers were built in their slab configuration. This slab was separated by a 15 Å vacuum layer in the *z* direction between the slab and its periodic images. A 3×3×1 Monkhorst-Pack *k*-point grid for Brillouin zone sampling was used in structural optimization and energy calculation. In order to fully consider the activity of each catalyst, the possible catalytic sites in catalysts are selected to discuss their adsorption behavior. A grid-based decomposition scheme of the electron density was used to perform the Bader charge analysis.

5. Supporting Figures

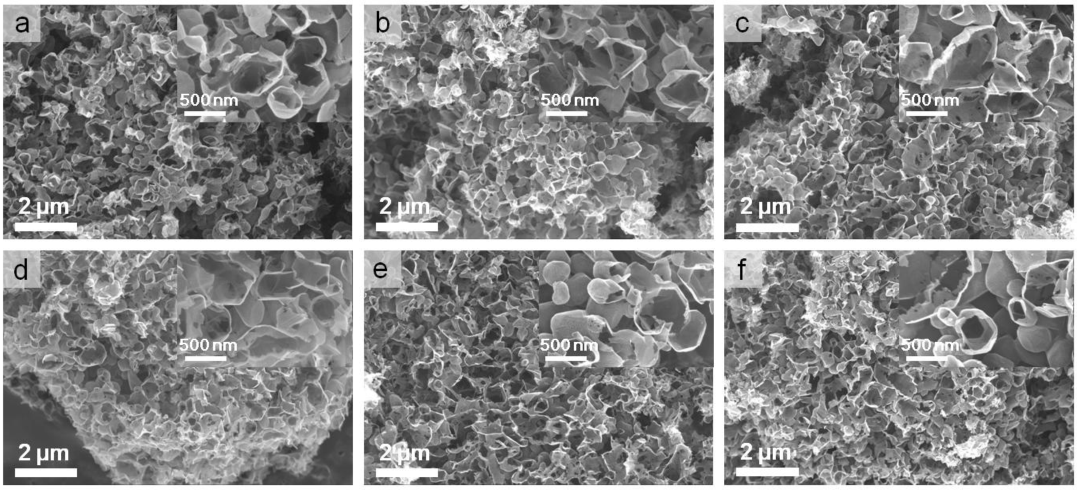

Figure S1. SEM images of (a) HPCS, (b) Fe_2O_3/HPCS, (c) RuO_2/HPCS, (d) Fe_{10}-RuO_2/HPCS, (e) Fe_{15}-RuO_2/HPCS, (f) Fe_{20}-RuO_2/HPCS at different magnifications. The morphology of HPCS is basically consistent with that reported previously.[1-3]

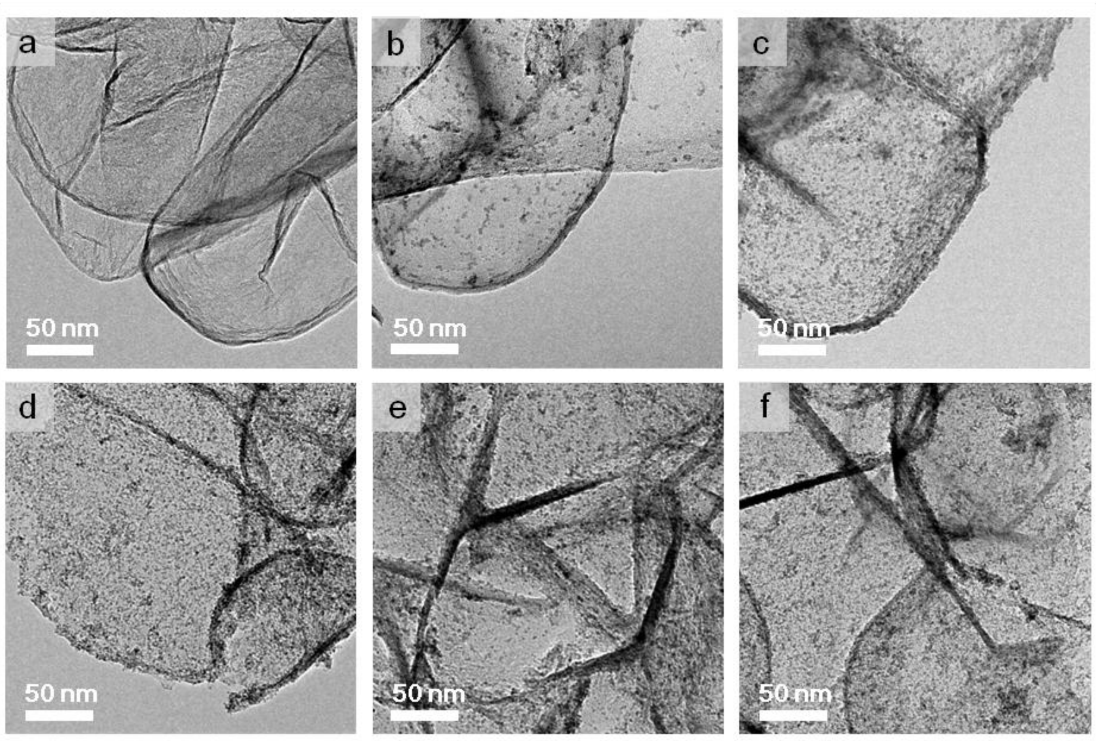

Figure S2. TEM images of (a) HPCS, (b) Fe_2O_3/HPCS, (c) RuO_2/HPCS, (d) Fe_{10}-RuO_2/HPCS, (e) Fe_{15}-RuO_2/HPCS, (f) Fe_{20}-RuO_2/HPCS.

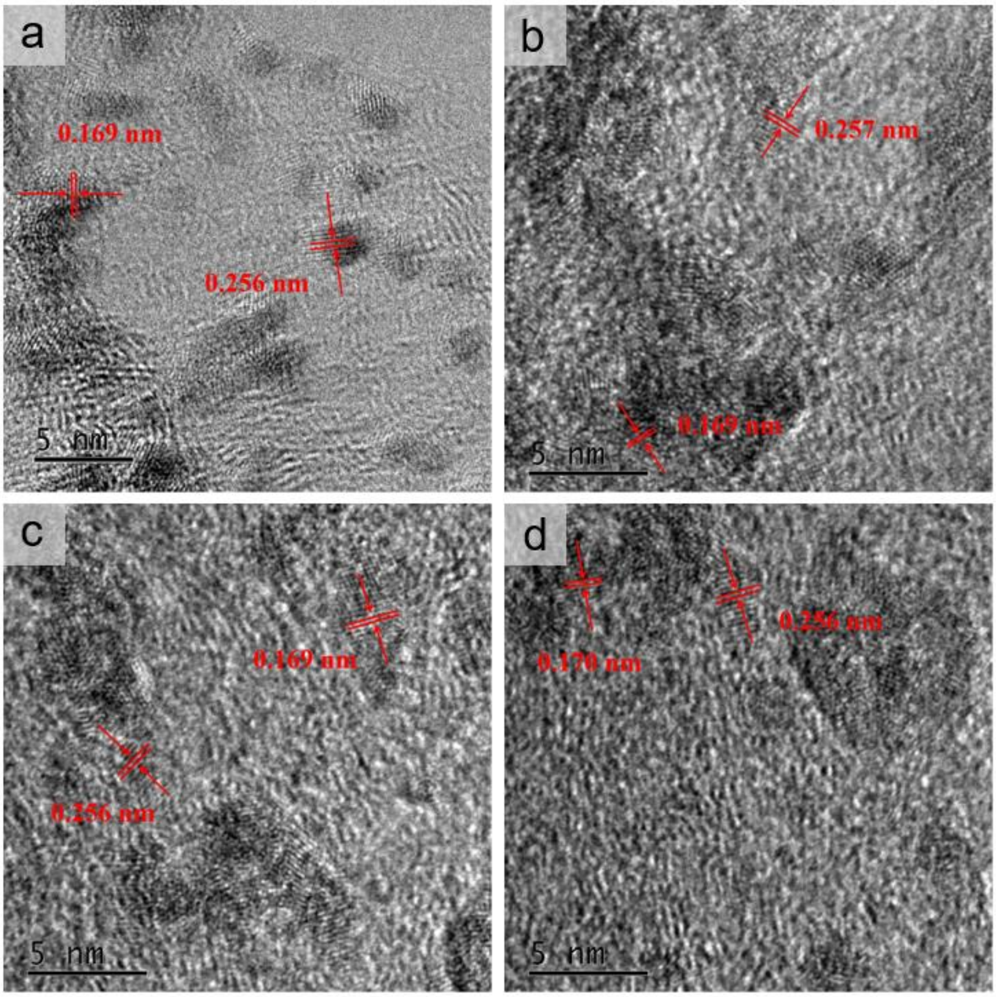

Figure S3. HRTEM images of (a) RuO_2/HPCS, (b) Fe_{10}-RuO_2/HPCS, (c) Fe_{15}-RuO_2/HPCS, (d) Fe_{20}-RuO_2/HPCS.

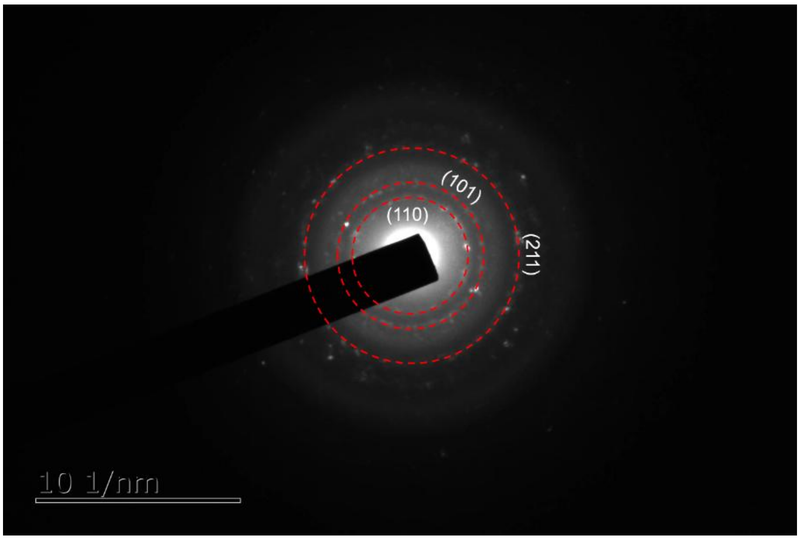

Figure S4. The selected-area electron diffraction (SAED) result of Fe_{SA}-RuO_2/HPCS sample, these diffraction rings are attributed to the (110), (101) and (211) crystal planes of rutile RuO_2, respectively.

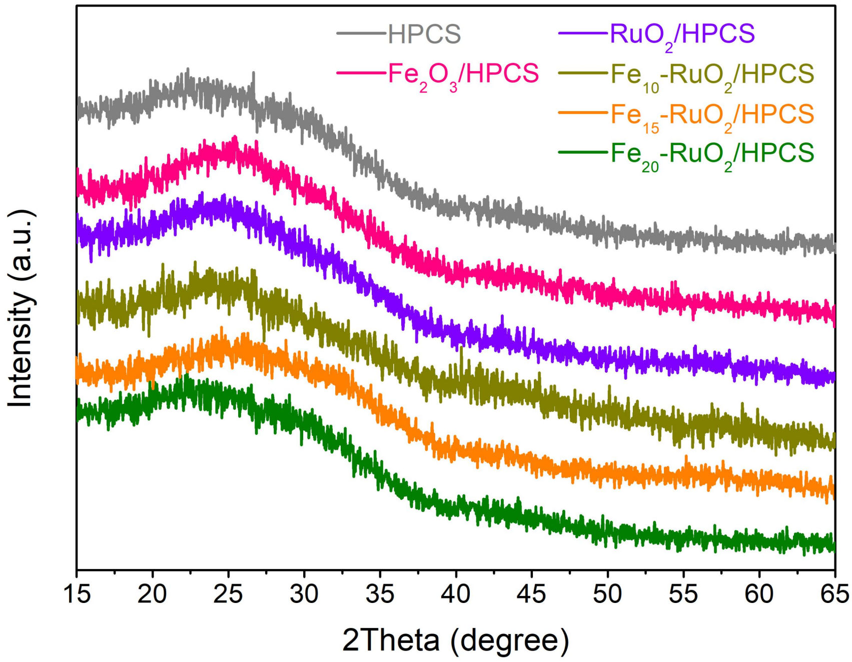

Figure S5. XRD patterns of HPCS, Fe_2O_3/HPCS, RuO_2/HPCS, Fe_{10}-RuO_2/HPCS, Fe_{15}-RuO_2/HPCS, Fe_{20}-RuO_2/HPCS samples.[1-3]

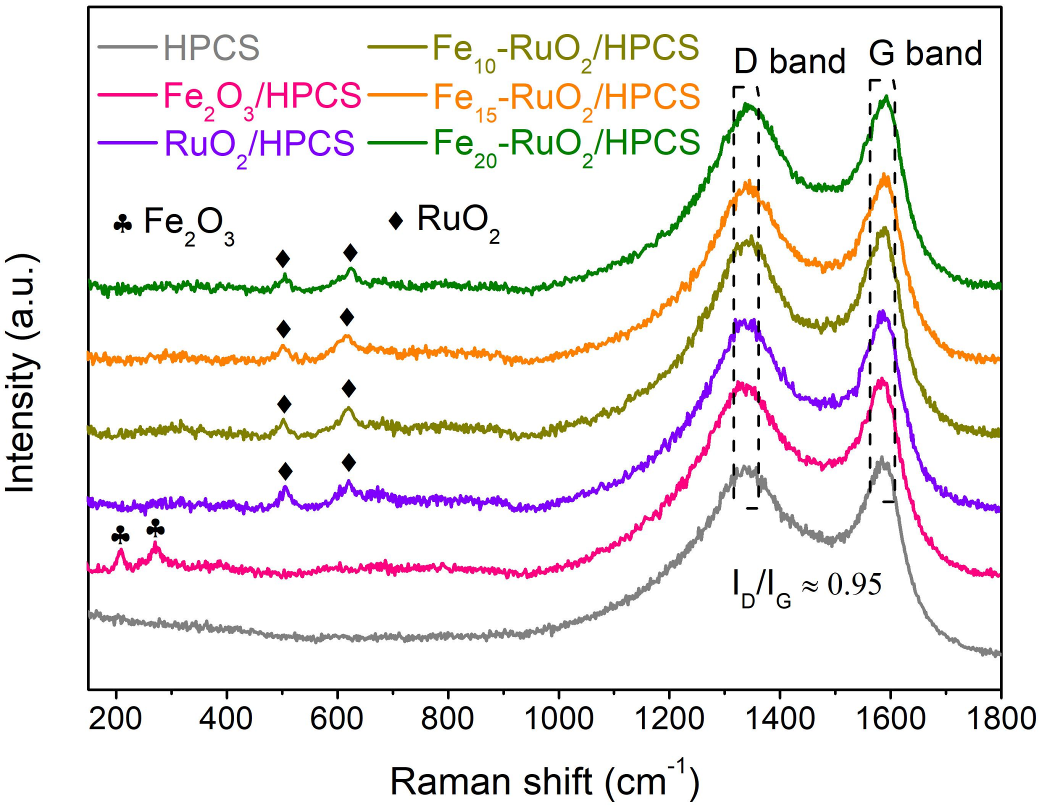

Figure S6. Raman spectra of HPCS, Fe_2O_3/HPCS, RuO_2/HPCS, Fe_{10}-RuO_2/HPCS, Fe_{15}-RuO_2/HPCS, Fe_{20}-RuO_2/HPCS samples.[10,11]

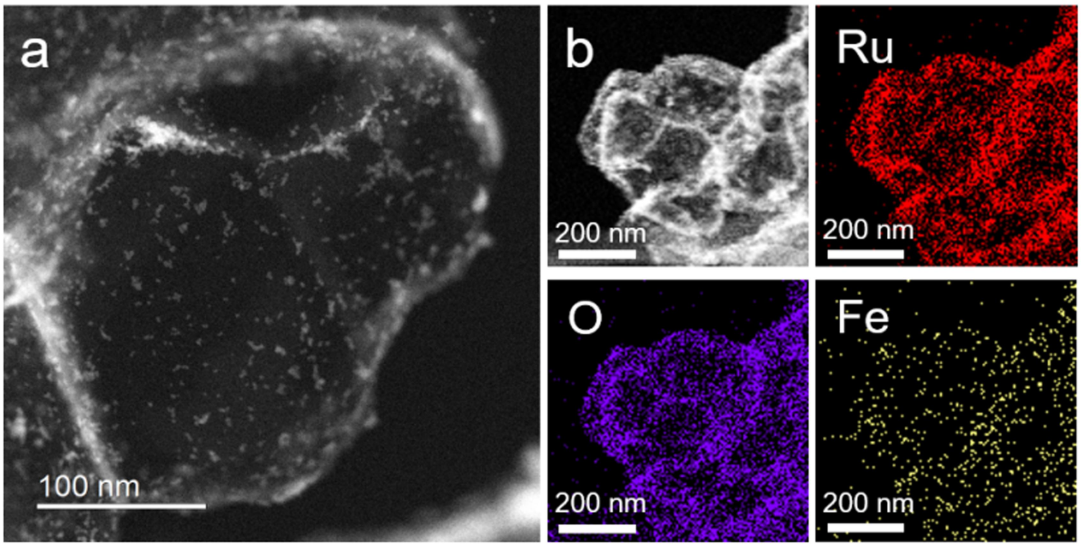

Figure S7. (a) HAADF-STEM image of Fe_{SA}-RuO_2/HPCS sample. (b) STEM image and EDX elemental mapping of Fe_{SA}-RuO_2/HPCS at a low-magnification.

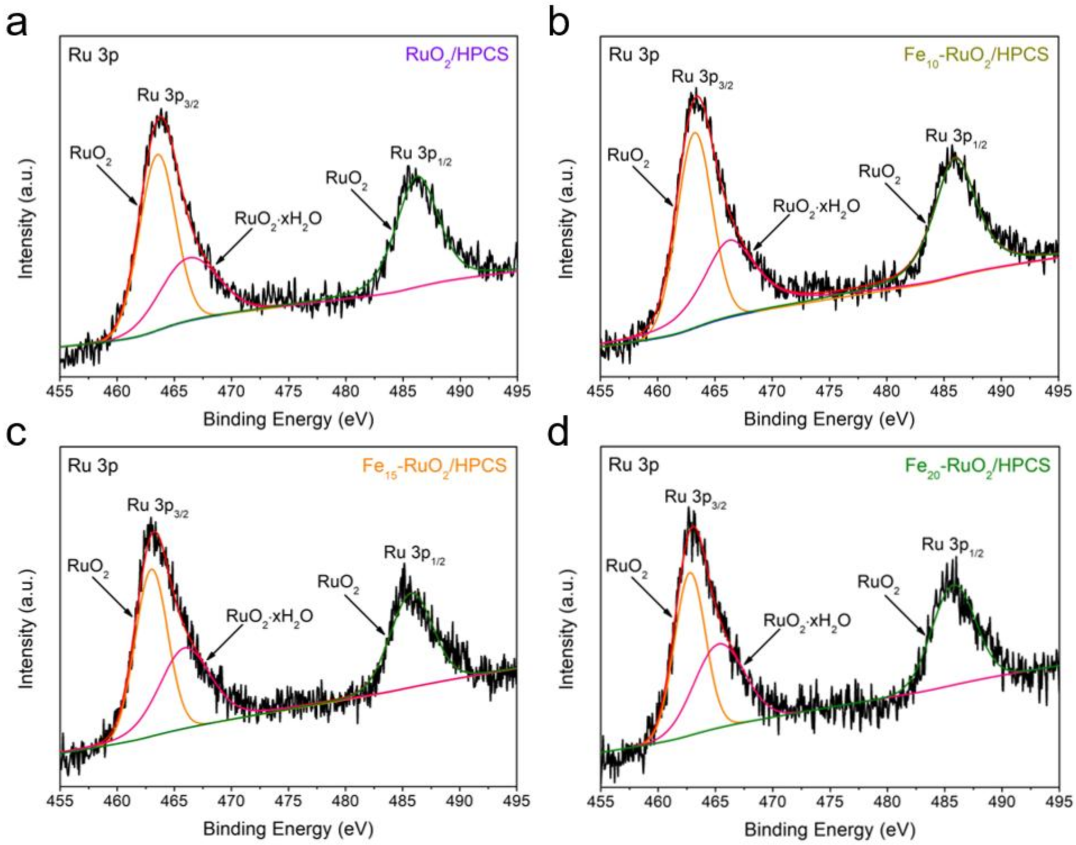

Figure S8. High-resolution XPS Ru 3p spectra of (a) RuO_2/HPCS, (b) Fe_{10}-RuO_2/HPCS, (c) Fe_{15}-RuO_2/HPCS, (d) Fe_{20}-RuO_2/HPCS samples.

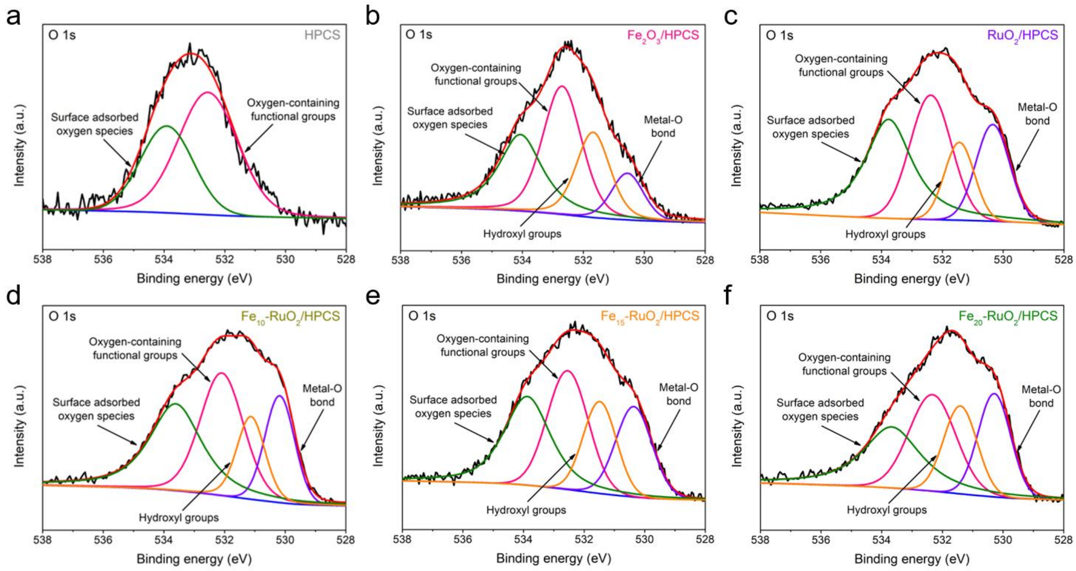

Figure S9. High-resolution XPS O 1s spectra of (a) HPCS, (b) Fe_2O_3/HPCS, (c) RuO_2/HPCS, (d) Fe_{10}-RuO_2/HPCS, (e) Fe_{15}-RuO_2/HPCS, (f) Fe_{20}-RuO_2/HPCS samples.

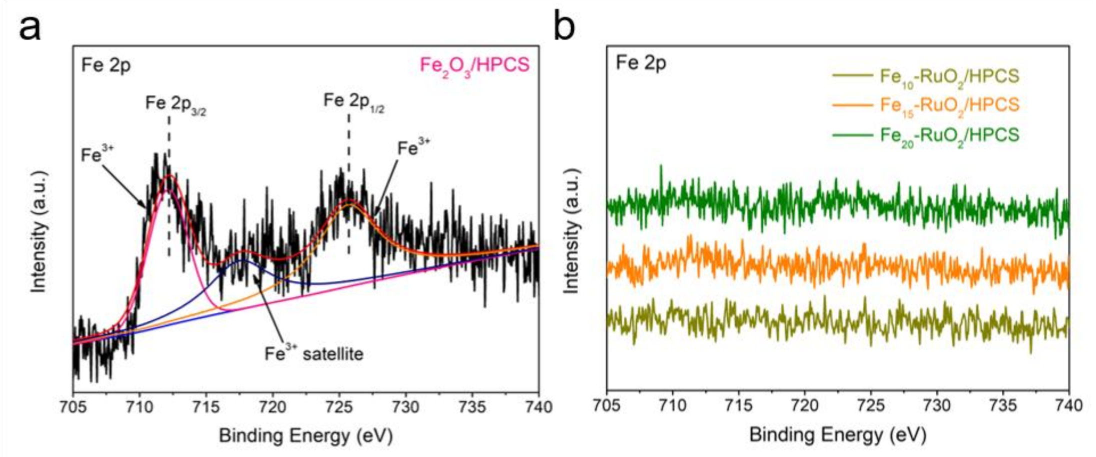

Figure S10. High-resolution XPS Fe 2p spectra of (a) Fe_2O_3/HPCS, (b) Fe_{10}-RuO_2/HPCS, Fe_{15}-RuO_2/HPCS and Fe_{20}-RuO_2/HPCS samples.

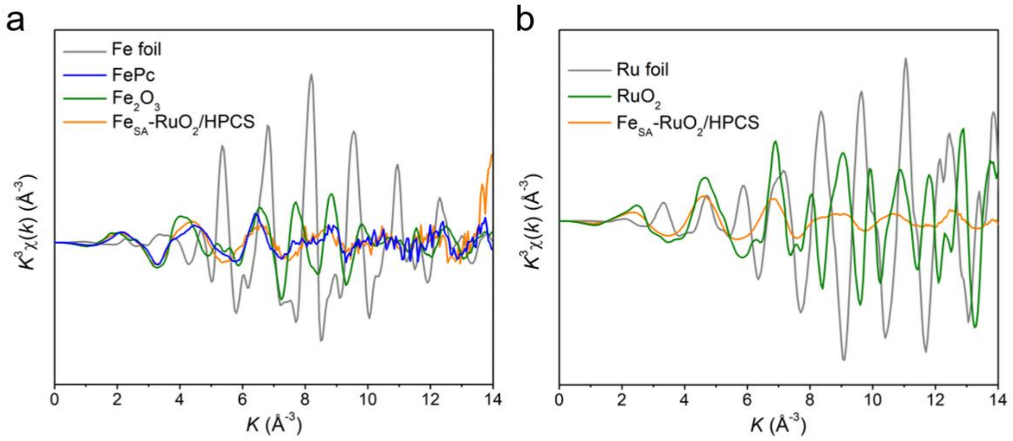

Figure S11. Fourier-transformed magnitudes of (a) Fe K-edge and (b) Ru K-edge EXAFS spectra in K space for Fe_{SA}-RuO_2/HPCS.

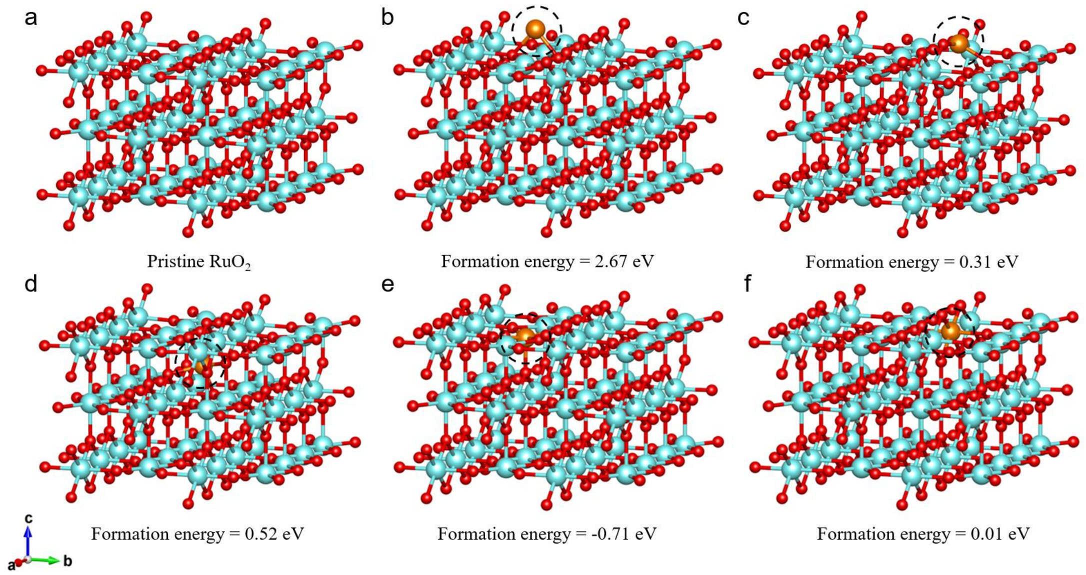

Figure S12. (a) Crystal model of pristine RuO_2. (b,c) Formation energies of Fe atom adsorbed at different positions on the surface of RuO_2 lattice. (d) Formation energy of Fe atom embedded in the interior of RuO_2 lattice. (e,f) Formation energies of Fe atom replacing different positions on the surface of RuO_2 lattice.

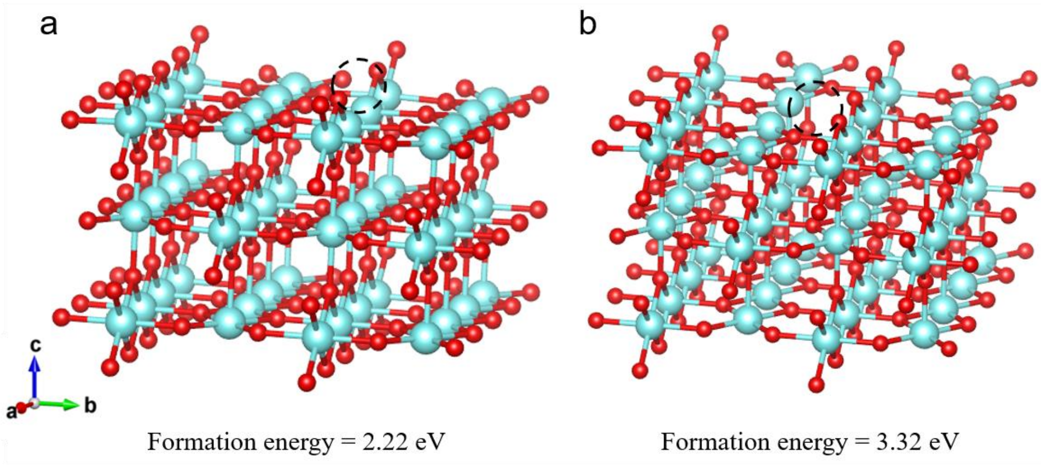

Figure S13. Oxygen vacancy formation energies at different positions on the surface of RuO_2 lattice.

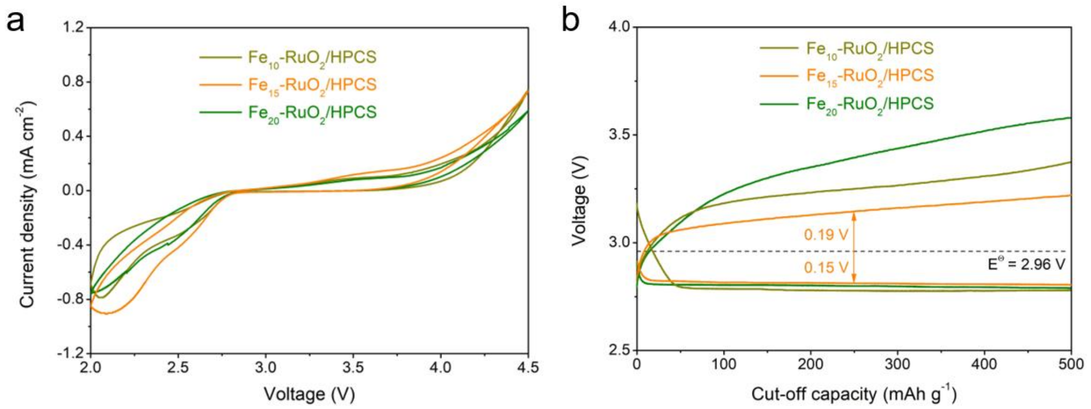

Figure S14. (a) CV curves of Fe_{10}-RuO_2/HPCS, Fe_{15}-RuO_2/HPCS and Fe_{20}-RuO_2/HPCS cathodes at a scan rate of 0.1 mV s^{-1} at a voltage window of 2.0-4.5 V. (b) Discharge-charge curves of Fe_{10}-RuO_2/HPCS, Fe_{15}-RuO_2/HPCS and Fe_{20}-RuO_2/HPCS cathodes at a curtailed capacity of 500 mAh g^{-1} at a current density of 100 mA g^{-1}.

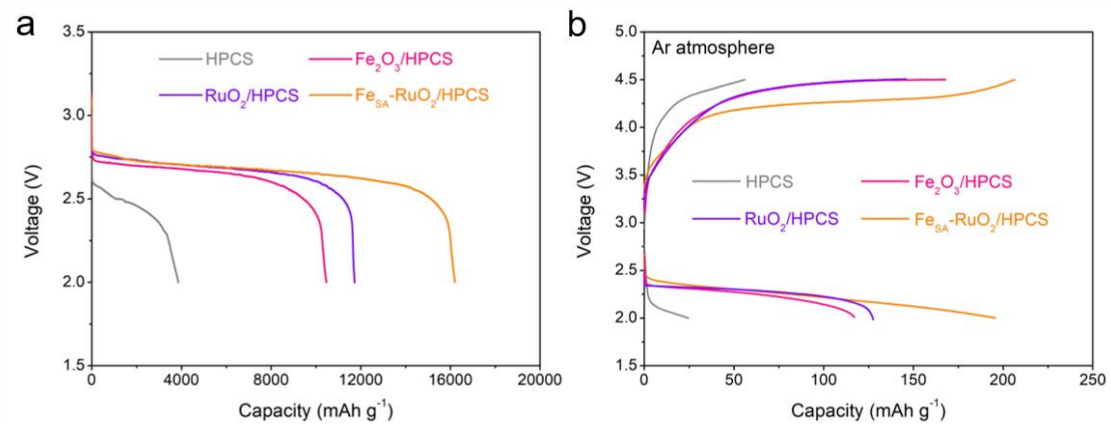

Figure S15. (a) Initial deep discharge curves of different cathodes at a current density of 300 mA g^{-1}. (b) When in an Ar atmosphere, the initial deep discharge-charge curves of batteries based on different cathodes at a current density of 200 mA g^{-1}.

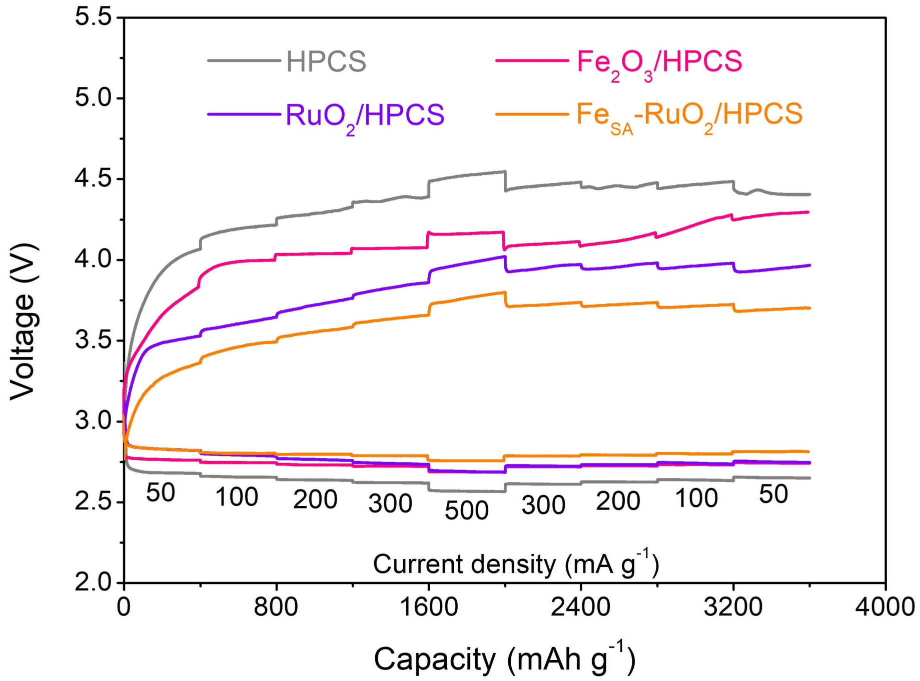

Figure S16. The rate performance of batteries based on HPCS, Fe_2O_3/HPCS, RuO_2/HPCS and Fe_{SA}-RuO_2/HPCS cathodes, the current density ranges from 50 to 500 mA g^{-1}.

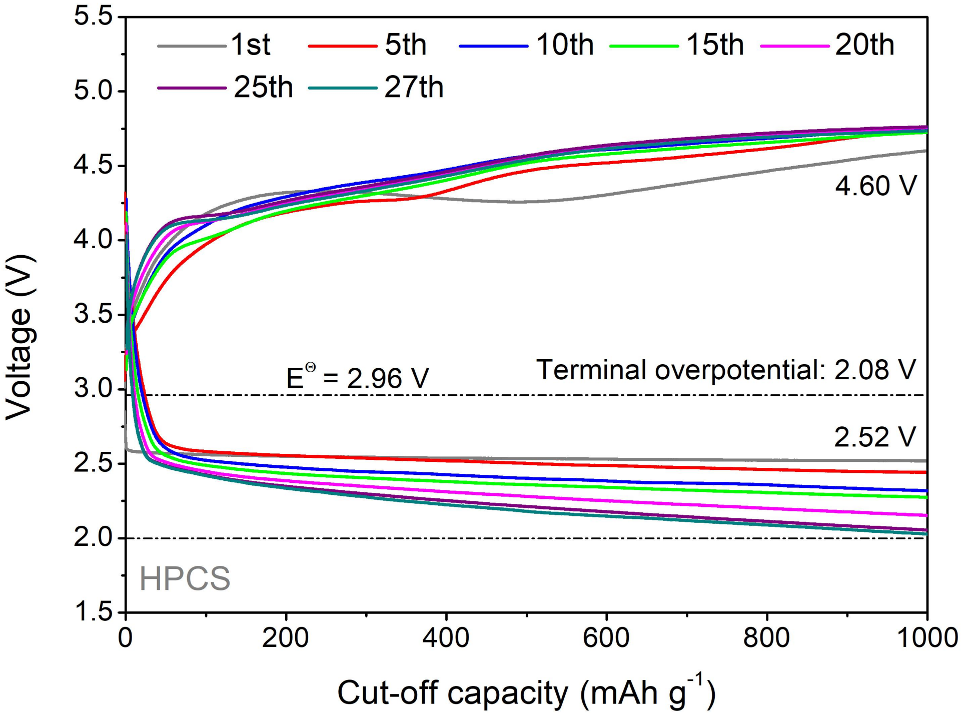

Figure S17. Discharge-charge profiles of HPCS cathodes with different cycles at 200 mA g^{-1} and 1000 mAh g^{-1}.

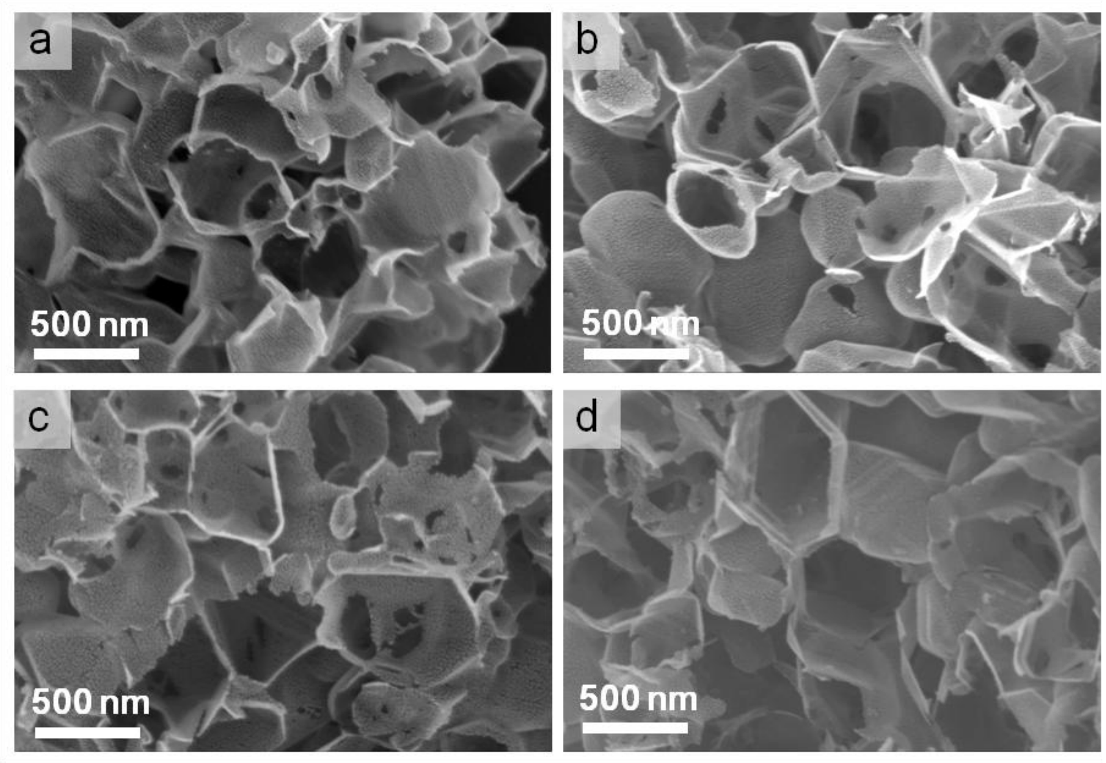

Figure S18. SEM images of pristine (a) HPCS, (b) Fe_2O_3/HPCS, (c) RuO_2/HPCS, (d) Fe_{SA}-RuO_2/HPCS cathodes.

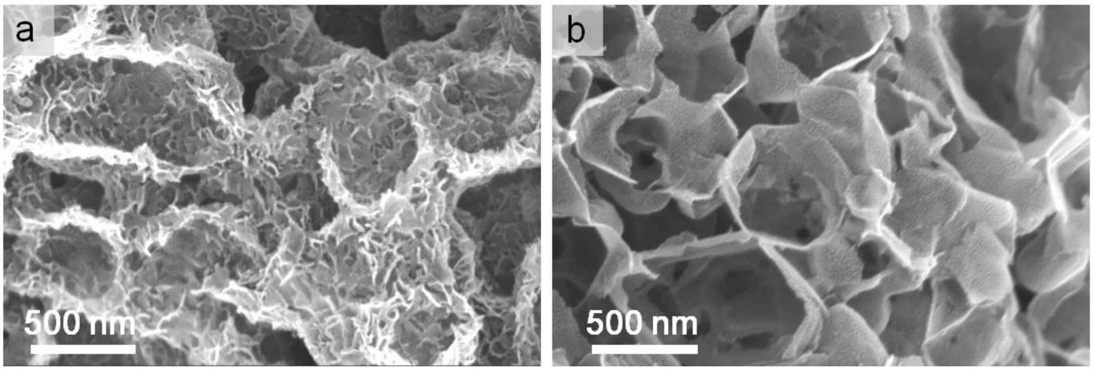

Figure S19. SEM images of Fe_2O_3/HPCS cathodes after (a) discharged and (b) recharged.

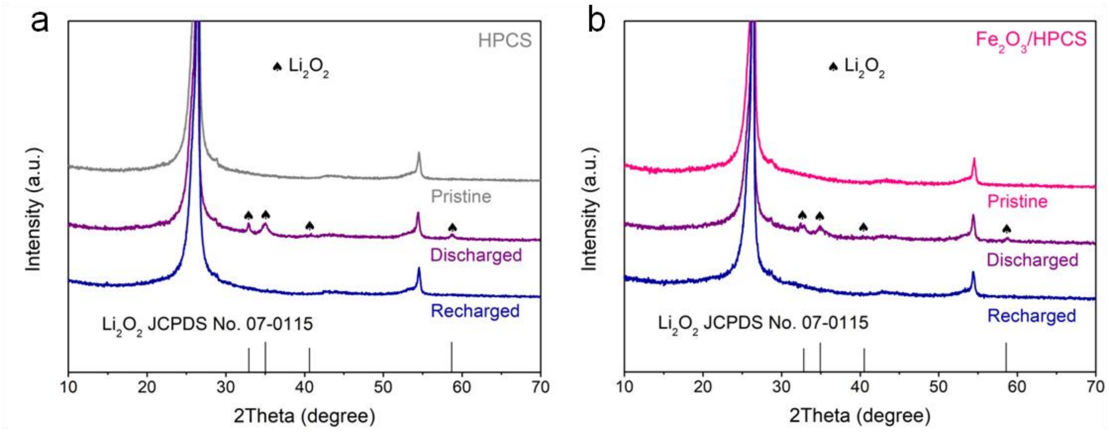

Figure S20. XRD patterns of pristine, discharged and recharged (a) HPCS and (b) Fe_2O_3/HPCS cathodes.

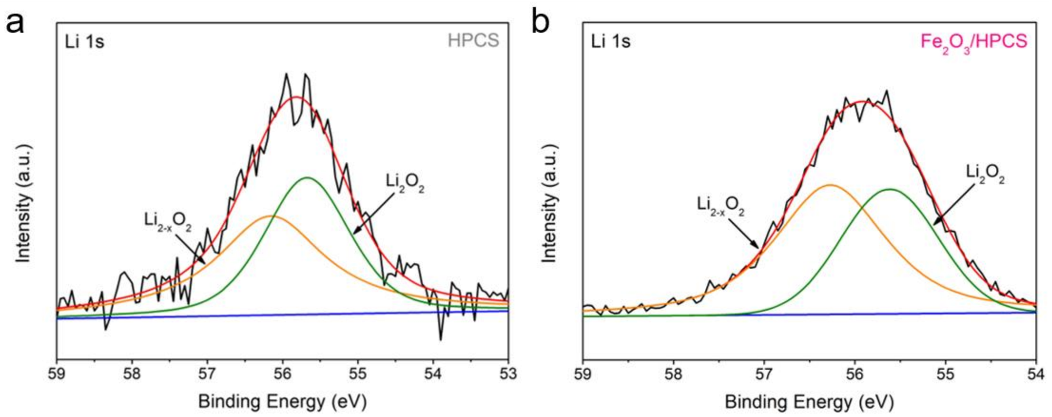

Figure S21. High-resolution Li 1s XPS spectra of (a) HPCS and (b) Fe_2O_3/HPCS cathodes after discharged.

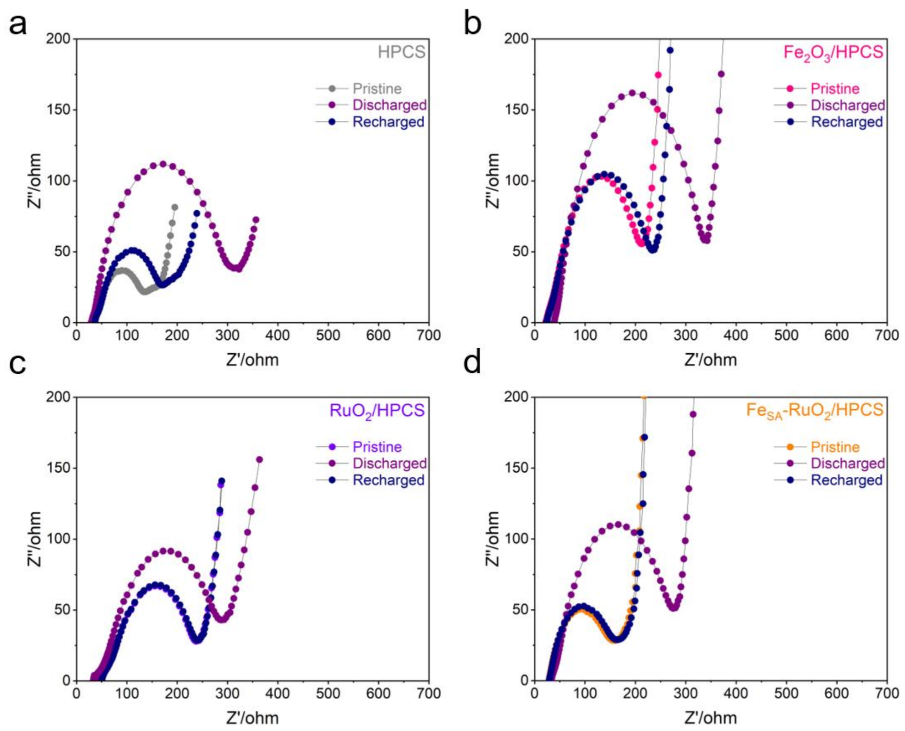

Figure S22. Electrochemical impedance spectra of LOBs with different cathodes catalyst at different stages.

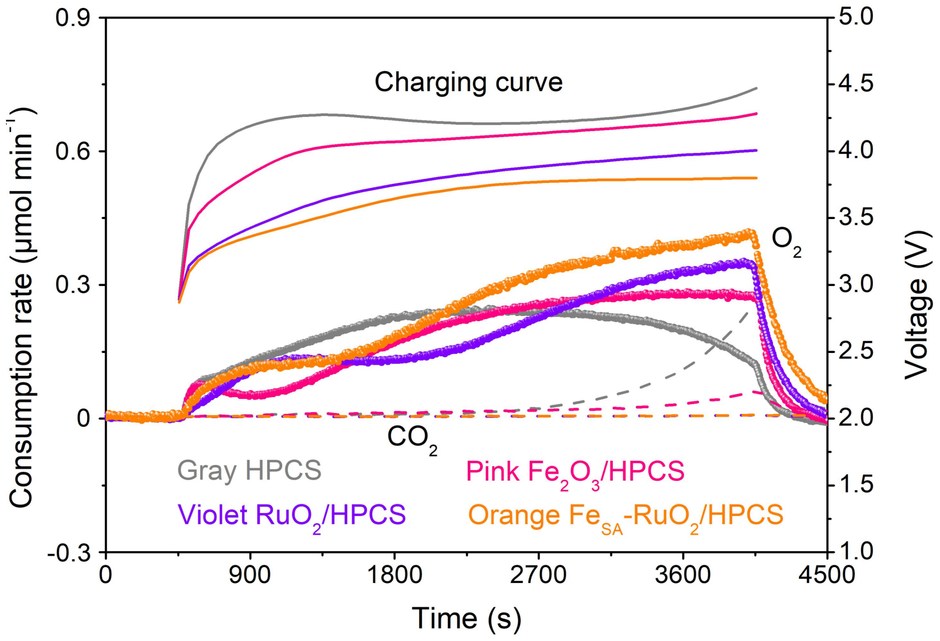

Figure S23. DEMS test results of LOBs based on HPCS, Fe_2O_3/HPCS, RuO_2/HPCS, Fe_{SA}- RuO_2/HPCS cathodes. Test conditions: 300 μA and 300 μAh.

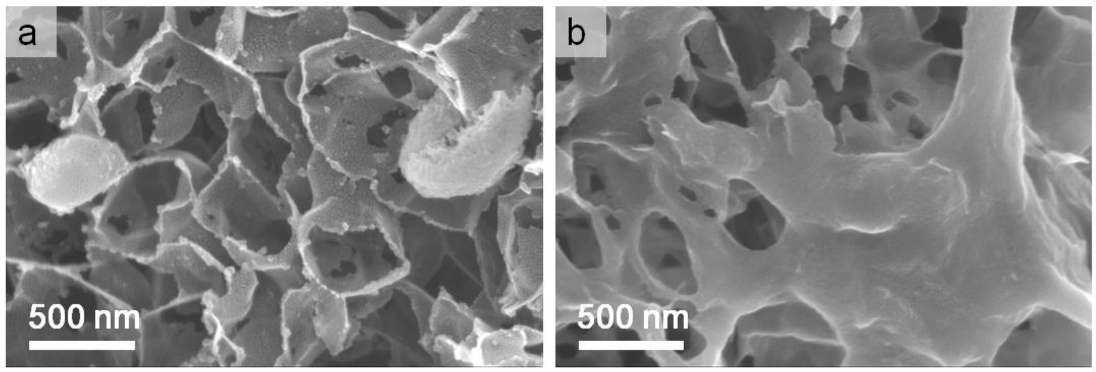

Figure S24. SEM images of HPCS cathodes after (a) 10 and (b) 27 cycles.

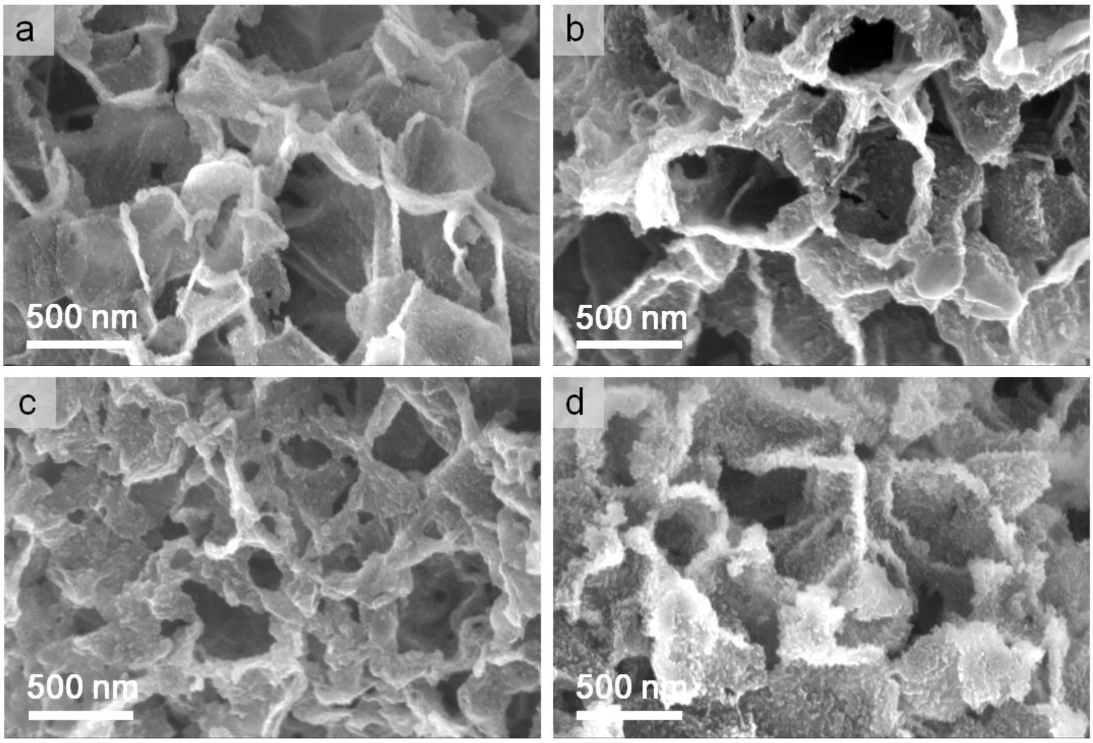

Figure S25. SEM images of Fe_2O_3/HPCS cathodes after (a) 10, (b) 30, (c) 50 and (d) 70 cycles.

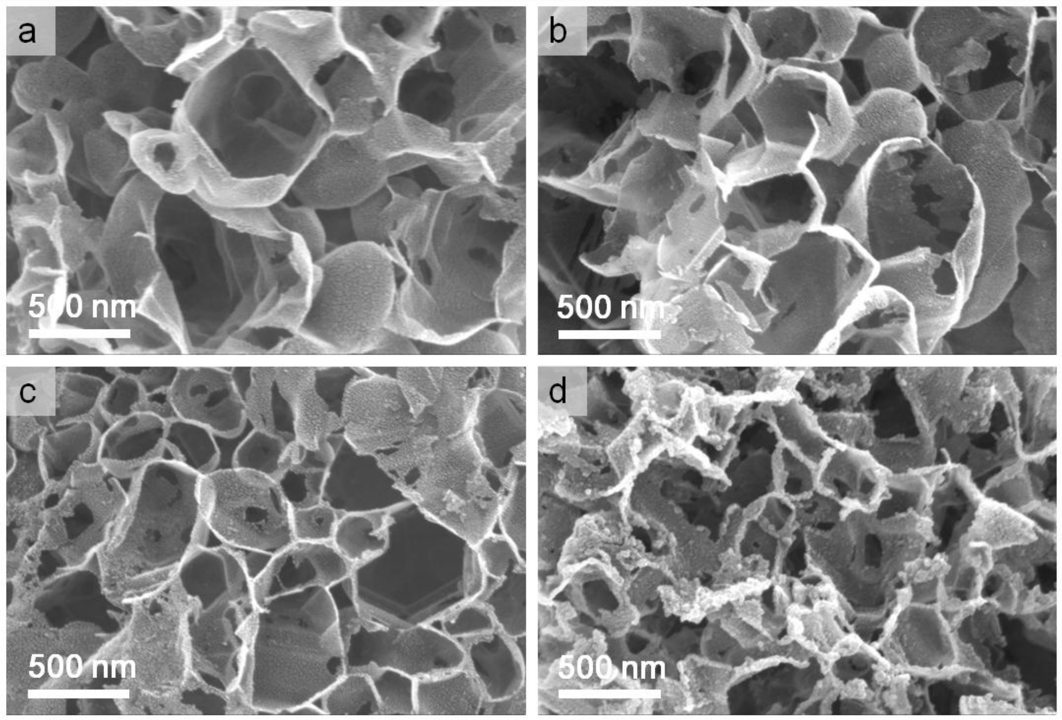

Figure S26. SEM images of RuO_2/HPCS cathodes after (a) 30, (b) 60, (c) 90 and (d) 134 cycles.

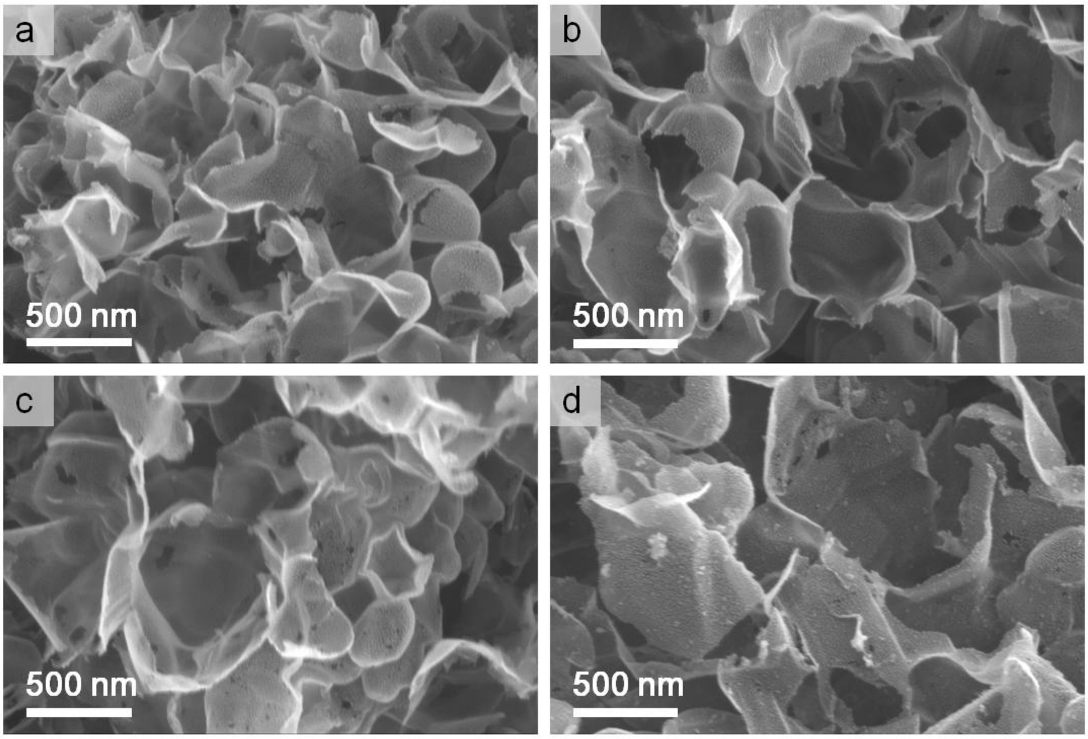

Figure S27. SEM images of Fe_{SA}-RuO_2/HPCS cathodes after (a) 60, (b) 120, (c) 180 and (d) 232 cycles.

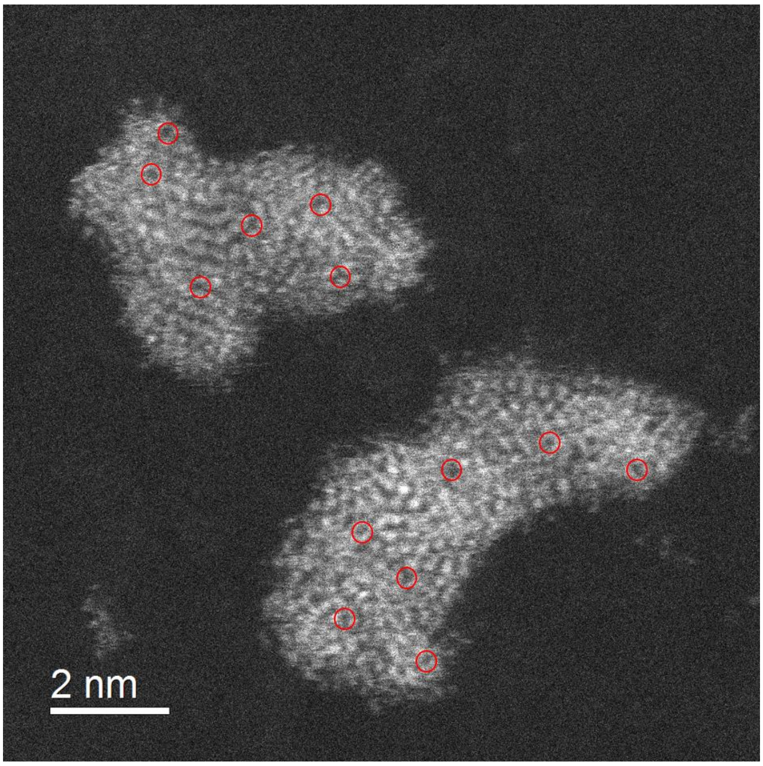

Figure S28. HAADF-STEM image of Fe_{SA}-RuO_2/HPCS cathode after 60 cycles.

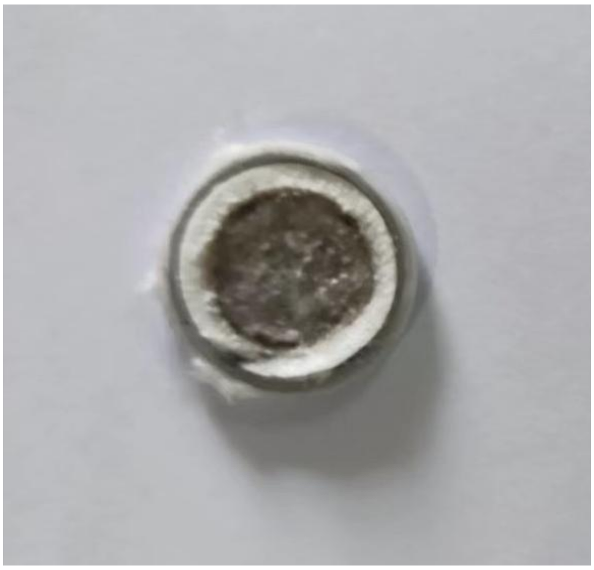

Figure S29. Digital image of the separator from the LOB assembled based on the Fe_{SA}-RuO_2/HPCS cathode after cycle failure. It is clear that the electrolyte has almost evaporated. At the same time, the black marks on the separator suggest severe degradation of the Li anode.

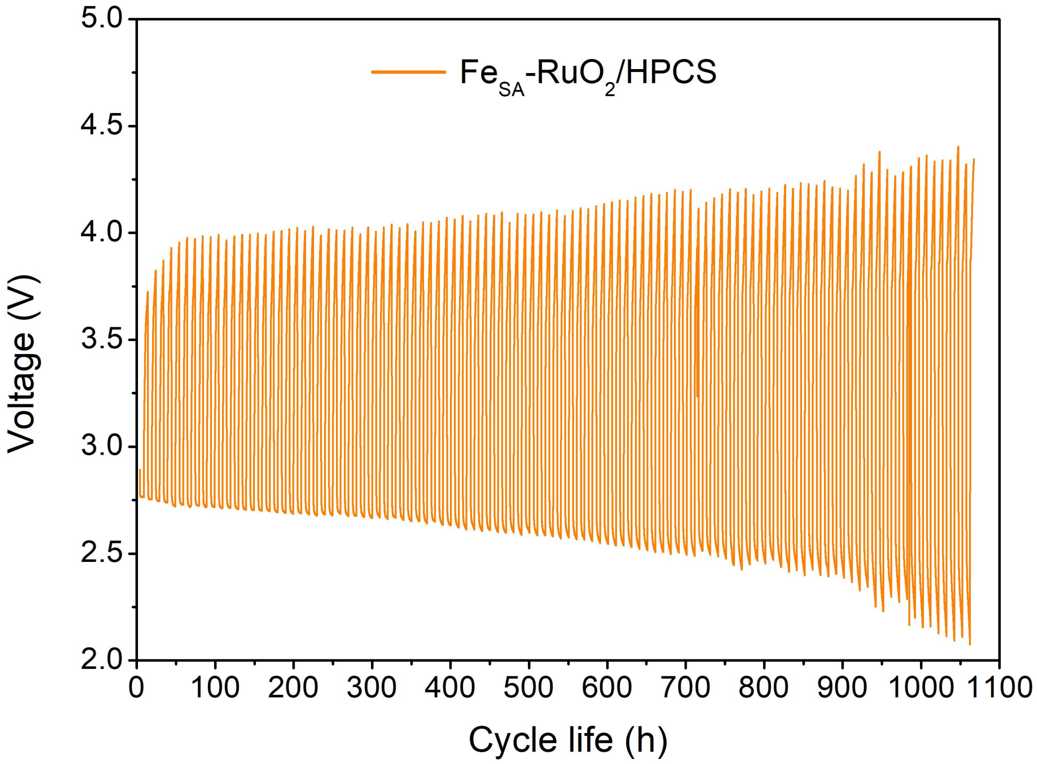

Figure S30. Cycle performance of LOB based on recycled Fe$_{SA}$-RuO$_2$/HPCS cathode.

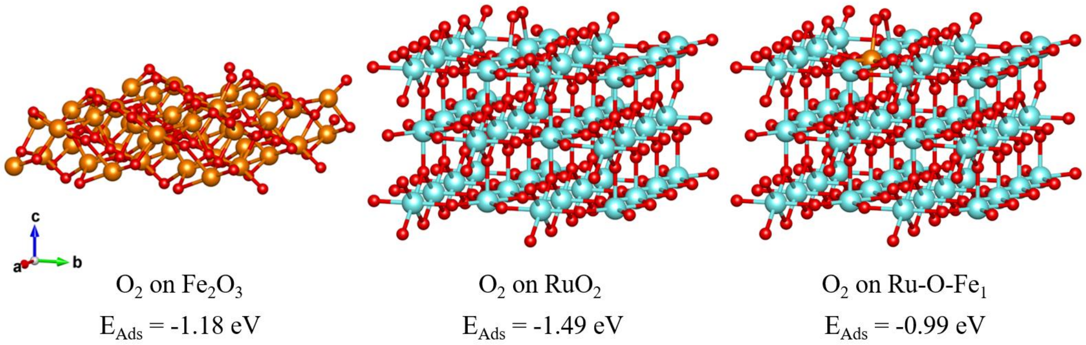

Figure S31. Adsorption energy of reaction gas O_2 on Fe_2O_3, RuO_2 and Ru-O-Fe_1 active sites.

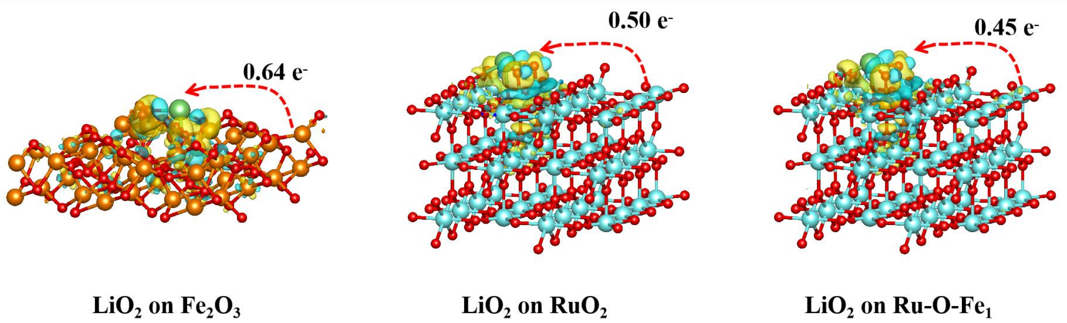

Figure S32. Charge density differences for LiO_2* adsorption states and corresponding charge transfer on the Fe_2O_3, RuO_2 and Ru-O-Fe_1 active sites.

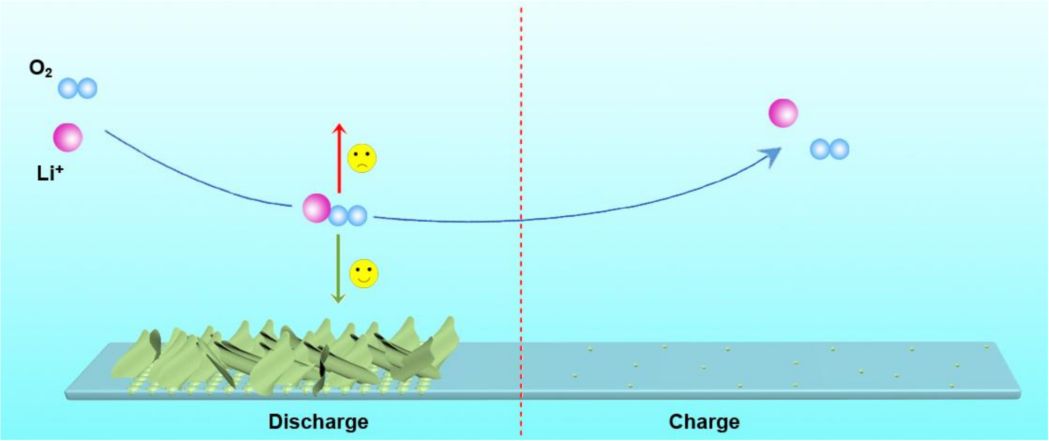

Figure S33. Schematic illustrations of the working mechanism for the Fe_2O_3/HPCS cathode.

6. Supporting Tables

Table S1. Fe and Ru elements content of different samples determined by ICP-OES.

Samples	Fe (wt. %)	Ru (wt. %)
Fe_2O_3/HPCS	4.61	0
RuO_2/HPCS	0	8.47
Fe_{10}-RuO_2/HPCS	0.14	8.51
Fe_{15}-RuO_2/HPCS	0.19	8.44
Fe_{20}-RuO_2/HPCS	0.23	8.46

Table S2. Various peak positions of high-resolution XPS Ru 3p spectra of RuO_2/HPCS, Fe_{10}-RuO_2/HPCS, Fe_{15}-RuO_2/HPCS, and Fe_{20}-RuO_2/HPCS samples.

Samples	Ru $3p_{1/2}$ (Ru^{4+})	Ru $3p_{3/2}$ (hydrated Ru oxide)	Ru $3p_{3/2}$ (Ru^{4+})
RuO_2/HPCS	486.23 eV	466.27 eV	463.52 eV
Fe_{10}-RuO_2/HPCS	486.08 eV	465.96 eV	463.39 eV
Fe_{15}-RuO_2/HPCS	485.96 eV	465.94 eV	463.21 eV
Fe_{20}-RuO_2/HPCS	485.77 eV	465.91 eV	462.99 eV

Table S3. EXAFS fitting parameters at the Fe K-edge and Ru K-edge for various samples.

Sample	Shell	*CN*	*R*(Å)	σ^2(Å^2)	ΔE_0 (eV)	*R* factor
Fe K-edge (S_0^2=0.745)						
Fe foil	Fe-Fe	8	2.47±0.01	0.0051±0.0010	6.7±1.6	0.0023
	Fe-Fe	6	2.85±0.01	0.0064±0.0019		
Sample Fe	Fe-O	5.4±0.4	1.95±0.01	0.0079±0.0010	-0.5±1.0	0.0073
Ru K-edge (S_0^2=0.807)						
Ru foil	Ru-Ru	12	2.67±0.01	0.0043±0.0003	3.3±0.9	0.0088
RuO_2	Ru-O	5.8±0.6	1.98±0.01	0.0029±0.0008	3.2±1.6	0.0068
	Ru-Ru	2.6±0.9	3.15±0.01	0.0031±0.0019	7.1±2.0	
	Ru-Ru	10.0±3.6	3.58±0.01	0.0031±0.0019		
Sample Ru	Ru-O	5.2±0.7	2.01±0.01	0.0070±0.0014	1.7±2.0	0.0128
	Ru-Fe	1.7±0.3	3.01±0.05	0.0106±0.0025		

CN, coordination number;
R, distance between absorber and backscatter atoms;
σ^2, Debye-Waller factor to account for both thermal and structural disorders;
ΔE_0, inner potential correction;
R factor indicates the goodness of the fit.
S_0^2 was fixed to 0.745 and 0.807.

Table S4. Comparison of electrochemical performances between Fe_{SA}-RuO_2/HPCS cathode and some RuO_2-based and single atom catalysts-based LOBs.

Cathode	Current density (mA g^{-1})	Cut-off capacity (mAh g^{-1})	Over-potential (V)	Discharge capacity (mAh g^{-1})	Cycle life (hour)	Reference
Mn_3O_4/CNTs-RuO_2 film	100	700	0.85	7198	>1700	12
RuO_2@LSCM NFs	50	500	1.32	12742	>1000	13
N-HMACs-RuO_2	200	1000	0.97	13400	2150	14
RuO_2@$NiCo_2O_4$	400	1000	~ 0.61	17633	>550	15
np-RuO_2/nr-MnO_2	50	500	0.58	>4000	>1400	16
RuO_2-MnO_2/MWCNTs	50	500	0.70	22000	>1800	17
RuO_2-Co_3O_4	200	500	0.84	19747	>500	18
Co SAs/N-C	200	1000	0.40	20105	>1300	19
$Ru_{0.3}$ SAs-NC	/	1000	0.55	13424	/	20
Ni_{SA}-Co_3O_4/CC	200	1000	0.72	21442	1280	21
SASe-Ti_3C_2	200	1000	1.10	17260	>1700	22
Ru_{NC}-Co_{SA}-3DNG	1000	1000	1.19	12362	>600	23
**Fe_{SA}-RuO_2/HPCS**	**200**	**1000**	**0.34**	**23628**	**2320**	**This work**

Supplementary References

[1] S. Y. Ma, Y. C. Lu, X. D. Zhu, Z. J. Li, Q. C. Liu, *ACS Appl. Mater. Interfaces* **2022**, 14, 22104.

[2] Z. Lian, Y. Pei, S. Y. Ma, Y. C. Lu, Q. C. Liu, *ChemistrySelect* **2022**, 7, e202104549.

[3] W. Yang, W. Yang, F. Ding, L. Sang, Z. P. Ma, G. J. Shao, *Carbon* **2017**, 111, 419.

[4] G. Kresse, J. Furthmüller, *Mater. Sci.* **1996**, 6, 15.

[5] G. Kresse, J. Furthmüller, *Phys. Rev. B* **1996**, 54, 11169.

[6] C. G. Hu, L. L. Gong, Y. Xiao, Y. F. Yuan, N. M. Bedford, Z. H. Xia, L. Ma, T. P. Wu, Y. Lin, J. W. Connell, R. Shahbazian-Yassar, J. Lu, K. Amine, L. M. Dai, *Adv. Mater.* **2022**, 32, 1907436.

[7] J. Perdew, K. Burke, M. Ernzerhof, *Phys. Rev. Lett.* **1996**, 77, 3865.

[8] L. L. Gong, L. X. W. Wang, T. Zheng, J. Liu, J. Wang, Y. C. Yang, J. Zhang, X. Han, L. P. Zhang, Z. H. Xia, *J. Mater. Chem. A* **2021**, 9, 3555.

[9] G. Kresse, D. Joubert, *Phys. Rev. B* **1999**, 59, 1758.

[10] H. Sun, W. Jung, *J. Mater. Chem. A* **2021**, 9, 15506.

[11] Z. Zhu, Q. L. Lv, Y. X. Ni, S. N. Gao, J. R. Geng, J. Liang, F. J. Li, *Angew. Chem., Int. Ed.* **2022**, 61, e202116699.

[12] C. T. Zhao, C. Yu, M. N. Banis, Q. Sun, M. D. Zhang, X. Li, Y. L. Liu, Y. Zhao, H. W. Huang, S. F. Li, X. T. Han, B. W. Xiao, Z. X. Song, R. Y. Li, J. S. Qiu, X. L. Sun, *Nano Energy* **2017**, 34, 399.

[13] X. L. Zhang, Y. D. Gong, S. Q. Li, C. W. Sun, *ACS Catal.* **2017**, 7, 7737.

[14] X. D. Zhu, Y. Shang, Y. C. Lu, C. M. Liu, Z. J. Li, Q. C. Liu, *J. Power Sources* **2020**, 471, 228444.

[15] L. Zou, Y. X. Zhang, J. F. Cheng, Y. Chen, B. Chi, J. Pu, L. Jian, *Electrochim. Acta* **2018**, 262, 97.

[16] Y. F. Xu, Y. Chen, G. L. Xu, X. R. Zhang, Z. H. Chen, J. T. Li, L. Huang, K. Amine, S. G. Sun, *Nano Energy* **2016**, 28, 63.

[17] C. S. Luo, H. Sun, Z. L. Jiang, H. L. Guo, M. Y. Gao, M. H. Wei, Z. M. Jiang, H. J. Zhou, S. G. Sun, *Electrochim. Acta* **2018**, 282, 56.

[18] Y. Zhang, S. T. Zhang, J. Ma, A. J. Huang, M. W. Yuan, Y. F. Li, G. B. Sun, C. Chen, C. Y. Nan, *ACS Appl. Mater. Interfaces* **2021**, 13, 39239.

[19] P. Wang, Y. Y. Ren, R. T. Wang, P. Zhang, M. J. Ding, C. X. Li, D. Y. Zhao, Z. Qian, Z. W. Zhang, L. Y. Zhang, L. W. Yin, *Nat. Commun.* **2020**, 11, 1576.

[20] X. L. Hu, G. Luo, Q. N. Zhao, D. Wu, T. X. Yang, J. Wen, R. H. Wang, C. H. Xu, N. Hu, *J. Am. Chem. Soc.* **2020**, 142, 16776.

[21] Z. Lian, Y. C. Lu, S. Y. Ma, Z. J. Li, Q. C. Liu, *Chem. Eng. J.* **2022**, 445, 136852.

[22] D. Y. Zhao, P. Wang, H. X. Di, P. Zhang, X. B. Hui, L. W. Yin, *Adv. Funct. Mater.* **2021**, 31, 2010544.

[23] M. R. Liu, J. Li, B. Chi, L. Zheng, Y. X. Zhang, Q. H. Zhang, T. Tang, L. R. Zheng, S. J. Liao, *J. Mater. Chem. A* **2021**, 9, 10747.
